# Mammalian-like type II glutaminyl cyclases in *Porphyromonas gingivalis* and other oral pathogenic bacteria as targets for treatment of periodontitis

**DOI:** 10.1016/j.jbc.2021.100263

**Published:** 2021-01-08

**Authors:** Nadine Taudte, Miriam Linnert, Jens-Ulrich Rahfeld, Anke Piechotta, Daniel Ramsbeck, Mirko Buchholz, Petr Kolenko, Christoph Parthier, John A. Houston, Florian Veillard, Sigrun Eick, Jan Potempa, Stephan Schilling, Hans-Ulrich Demuth, Milton T. Stubbs

**Affiliations:** 1 Periotrap Pharmaceuticals GmbH, Halle (Saale), Germany; 2 Department of Molecular Drug Design and Target Validation, Fraunhofer Institute for Cell Therapy and Immunology, Halle (Saale), Germany; 3 Institut für Biochemie und Biotechnologie, Charles-Tanford-Proteinzentrum, Martin-Luther-Universität Halle-Wittenberg, Halle (Saale), Germany; 4 Department of Oral Immunology and Infectious Diseases, School of Dentistry, University of Louisville, Louisville, Kentucky, USA; 5 Faculty of Biochemistry, Biophysics and Biotechnology, Jagiellonian University, Krakow, Poland; 6 Department of Periodontology, School of Dental Medicine, University of Bern, Bern, Switzerland; 7 Angewandte Biowissenschaften und Prozesstechnik, Hochschule Anhalt, Köthen, Germany; 8 ZIK HALOmem, Charles-Tanford-Proteinzentrum, Martin-Luther-University Halle-Wittenberg, Halle (Saale), Germany

**Keywords:** bacterial pathogens, crystal structure, drug design, enzyme structure, periodontal disease, glutaminyl cyclase, *Porphyromonas gingivalis*, ACAP, auto-antibodies against citrullinated peptides, AD, Alzheimer's disease, FRIL, freeze-fracture replica immunolabeling, GnRH, gonadotropin-releasing hormone, NMM, N-methylmorpholine, ORF, open reading frame, PPAD, *P. gingivalis* peptidylarginine deiminase, TRH, thyrotropin-releasing hormone

## Abstract

The development of a targeted therapy would significantly improve the treatment of periodontitis and its associated diseases including Alzheimer’s disease, rheumatoid arthritis, and cardiovascular diseases. Glutaminyl cyclases (QCs) from the oral pathogens *Porphyromonas gingivalis*, *Tannerella forsythia*, and *Prevotella intermedia* represent attractive target enzymes for small-molecule inhibitor development, as their action is likely to stabilize essential periplasmic and outer membrane proteins by N-terminal pyroglutamination. In contrast to other microbial QCs that utilize the so-called type I enzymes, these oral pathogens possess sequences corresponding to type II QCs, observed hitherto only in animals. However, whether differences between these bacteroidal QCs and animal QCs are sufficient to enable development of selective inhibitors is not clear. To learn more, we recombinantly expressed all three QCs. They exhibit comparable catalytic efficiencies and are inhibited by metal chelators. Crystal structures of the enzymes from *P. gingivalis* (*Pg*QC) and *T. forsythia* (*Tf*QC) reveal a tertiary structure composed of an eight-stranded β-sheet surrounded by seven α-helices, typical of animal type II QCs. In each case, an active site Zn ion is tetrahedrally coordinated by conserved residues. Nevertheless, significant differences to mammalian enzymes are found around the active site of the bacteroidal enzymes. Application of a *Pg*QC-selective inhibitor described here for the first time results in growth inhibition of two *P. gingivalis* clinical isolates in a dose-dependent manner. The insights gained by these studies will assist in the development of highly specific small-molecule bacteroidal QC inhibitors, paving the way for alternative therapies against periodontitis and associated diseases.

Periodontitis is a widespread bacterially driven chronic inflammatory disease of mankind, with an overall prevalence of 11.2% and around 743 million people affected worldwide (1). The disease has been characterized as a microbial shift-disease, where pathogenic bacteria of the oral microbiome become predominant (2, 3, 4). In particular, the keystone pathogen *Porphyromonas gingivalis* (5) together with other anaerobic bacteria such as *Tannerella forsythia* initiates dysbiosis, resulting in disruption of tissue homeostasis and normal immune response. This inadequate inflammatory host response leads finally to degradation of periodontal tissue (3, 4, 6)

Periodontitis affects not only the oral cavity; a number of studies have demonstrated links between periodontitis and systemic diseases, including cancer, cardiovascular disease, rheumatoid arthritis, and Alzheimer's disease (AD) (7, 8, 9). For example, high levels of *P. gingivalis* DNA have been detected in both synovial fluid and tissue of rheumatoid arthritis sufferers (10, 11), where it is thought that the *P. gingivalis* peptidylarginine deiminase (PPAD) catalyzes protein citrullination, stimulating the production of auto-antibodies against citrullinated peptides (ACAP) and thereby influencing the etiology of the disease (9).

Following the first detection of *P. gingivalis* in the brain tissue of AD patients (12), the relationship between AD and periodontitis has become a focus for research (7, 13, 14, 15, 16). Subsequent studies have revealed a correlation between *P. gingivalis* infection and the etiopathogenesis of Alzheimer's disease, presumably by activation of inflammatory mechanisms (13, 15, 16, 17, 18). Oral application of *P. gingivalis* in a transgenic mouse model of AD resulted in an increased level of proinflammatory cytokines Il-1β, TNF-α, and Aβ40/42 in the brain (16). Most recently, *P. gingivalis*-derived DNA and gingipains (caspase-like cysteine proteases characteristic of *P. gingivalis*) have been identified in the central nerve system of clinical AD patients (7). This study demonstrated that *P. gingivalis* infection of BALB/c mice results in brain infection as well as an increased Aβ 1-42 level, which can be prevented by treatment with gingipain-specific inhibitors, a finding that strongly suggests that gingipains play a central role in the pathogenesis of AD by inducing neuroinflammation and neurodegeneration.

Currently, periodontitis is treated by nonsurgical therapy such as debridement and the application of adjunctive antimicrobials, for example, chlorhexidine and the antibiotics minocycline, doxycycline, amoxicillin, or metronidazole (19, 20). As general antibiotic therapies can involve the risk of development of antibiotic-resistance bacteria and destruction of the host microbiome (leading in turn to a loss of metabolic support, immune modulation, and enabling recolonization by potential pathogens), an alternative therapy would be desirable, such as the selective small-molecule inhibition of a physiologically relevant bacterial enzyme.

The glutaminyl cyclase (QC) from *P. gingivalis* (*Pg*QC), identified recently using sophisticated proteomic analyses (21), represents such an attractive target. QCs belong to the family of aminoacyltransferases and catalyze the cyclization of N-terminal glutamine/glutamate residues of peptides and proteins with concomitant release of ammonia/water (Fig. 1*A*). They are of widespread distribution and can be found in mammals (22, 23), bacteria (24, 25), fungi (26), plants (27), and arthropods (28, 29). Although QCs of all kingdoms catalyze the same reaction, they differ concerning their structure and catalytic sites (30). Typically, bacterial and plant QCs exhibit a fivefold β-propeller structure and have been classified as type I QC enzymes (24, 25, 31) (Fig. 1*B*). In contrast, animals possess type II QCs, which exhibit a characteristic α/β hydrolase topology with a central catalytic, essential metal ion, and have been described so far only in mammals and arthropods (28, 29, 32) (Fig. 1*C*). Two isoforms are found in humans, one cytosolic (*Hs*QC) and one Golgi-resident (*Hs*isoQC), the latter of which possesses an N-terminal transmembrane helix (33), and *Drosophila melanogaster* possesses distinct cytosolic and mitochondrial isoforms (29).

**Figure 1:**
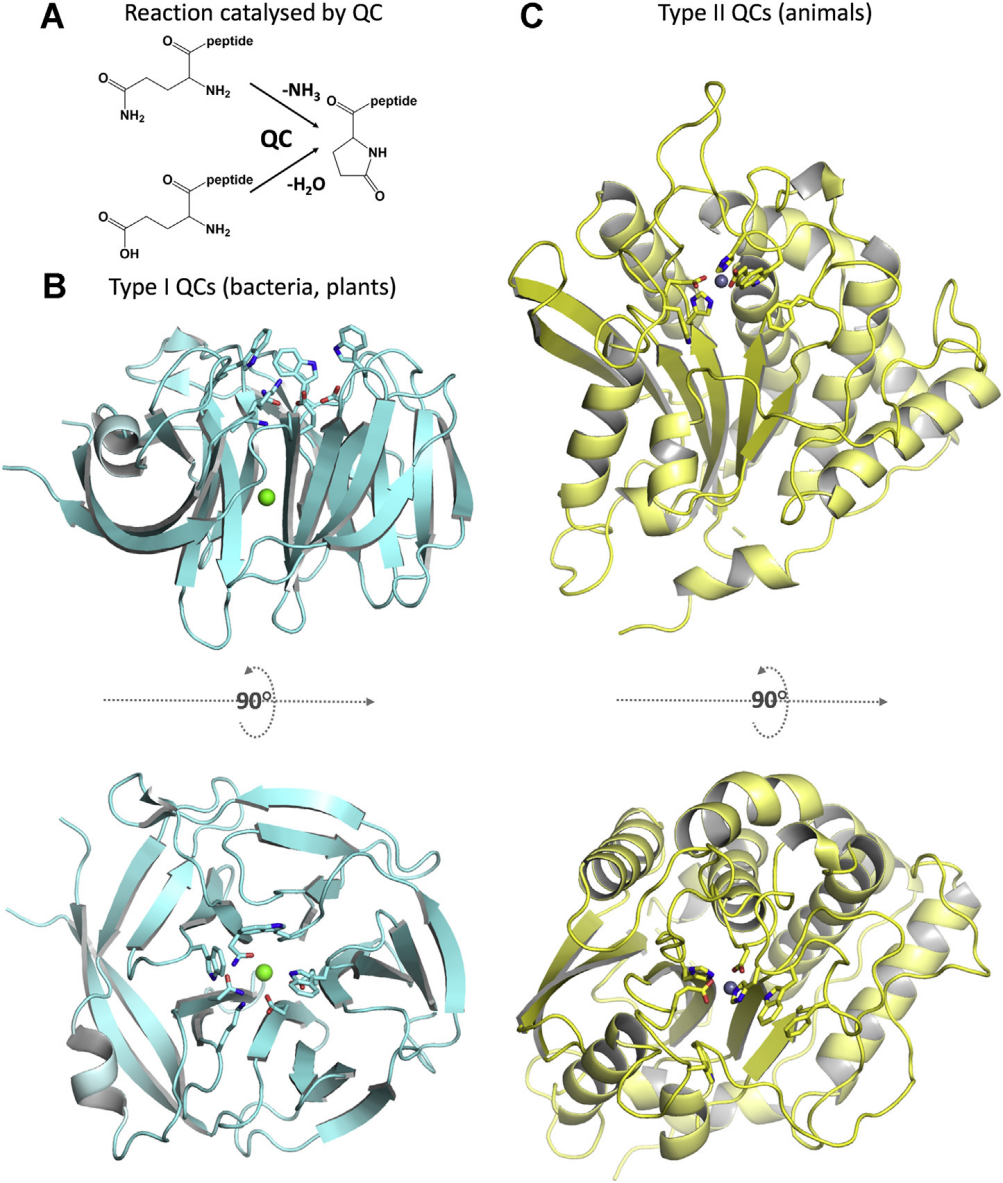
**Enzymes that cyclize N-terminal glutamine/glutamate residues segregate into distinct type I and type II QCs**. *A*, reaction catalyzed by QC enzymes. *B*, type I QCs, found in bacteria and plants, possess a five-bladed β-propeller structure (here the QC from *Zymomonas mobilis*, 3 nol). Residues at the active site are shown in stick representation; *green sphere* denotes the structural calcium ion. *C*, type II QCs, found until now only in mammals and arthropods, exhibit an α/β hydrolase fold, with a catalytic zinc ion (*gray sphere*) at the active site (human QC, 3 pbb).

The physiological role and targets of type I QCs are not fully known, whereas the type II human QC (*Hs*QC) catalyzes the N-terminal pyroglutamate formation of the chemokines CX3CL1 (fractalkine) and CCL2 (MCP-1, monocyte chemoattractant protein-1), of hormones such as thyrotropin-releasing hormone (TRH), gonadotropin-releasing hormone (GnRH), neurotensin, and gastrin and of collagen or fibronectin. In humans, introduction of this posttranslational modification has been shown to be essential for stability against N-terminal degradation and for modulation of receptor binding (23, 30, 33, 34, 35). In addition to these physiological substrates, *Hs*QC also cyclizes truncated Glu3-Aβ peptide, generating pGlu3-Aβ, a significant component of Aβ plaques in AD brains (34). The cyclization of the N-terminal residue increases the stability, hydrophobicity, aggregation potential, and thereby toxicity of the peptide (35). Because pGlu3-Aβ is one of the most abundant components of the plaque in AD brains, it appears that QC plays an important role in the development and progression of AD. For this reason, selective inhibition of QC activity is being explored in clinical phase II trials as a treatment option for AD (36).

Surprisingly, putative QC open reading frames (ORFs) from the order *Bacteroidales*, which includes the families Porphyromonadaceae, Bacteroidaceae and Prevotellaceae and the oral pathogens *P. gingivalis*, *T. forsythia*, and *Prevotella intermedia*, would appear to belong to type II (“animal”-type) QCs (21). Primary sequences of these gene products exhibit 25% (*Pg*QC) or 23% (*Tf*QC and *Pi*QC) sequence identity to human glutaminyl cyclase (*Hs*QC), including residues corresponding to the highly conserved metal binding motif in mammalian QCs (Asp159, Glu202, and His330 in *Hs*QC) (32); the zinc ligand *Hs*Glu202 is replaced by a conserved Asp residue (*Pg*Asp183) in the bacteroidal sequences. In addition, the latter possess counterparts to residues assumed to be involved in catalysis (*Hs*Glu201 and *Hs*Asp248) and to line the active site (*Hs*Phe325 and *Hs*Trp329). Further analyses indicated the presence of an N-terminal lipid anchor (37), consistent with the authors’ demonstration that *Pg*QC localizes to the inner periplasmatic membrane, which in turn is supported by freeze-fracture replica immunolabeling (FRIL) electron microscopy (38). It is thought that the enzyme catalyzes the cyclization of N-terminal glutamine residues of periplasmic, outer membrane integrated, and extracellular proteins after their translocation into the periplasm and subsequent removal of the signal peptides by the SP I signal peptidase (21, 39, 40), which could stabilize substrate proteins by protecting them against proteolytic degradation by periplasmatic and host cell aminopeptidases.

As such protein modifications could be important for the survival of *P. gingivalis* (by ensuring nutrient acquisition from the host, facilitating response to environmental changes, and/or delivering virulence factors), the catalytic action of QC may be beneficial for the overall physiological fitness of *P. gingivalis*. This is supported by saturation mariner transposon insertion sequencing of the genome of *P. gingivalis* ATCC 33277 by two independent groups, which identified *Pg*QC (PGN_0202) as one of 281 candidate essential genes (from ∼117.000 TA sites distributed randomly over 2155 genes in the whole genome) (41, 42).

Thus, the QC of *P. gingivalis* and other oral pathogens represent attractive targets for small-molecule inhibitor development for the treatment or prevention of chronic periodontitis. In this study, we characterize and compare enzymatic properties of three different bacteroidal QCs: from *P. gingivalis* (*Pg*QC), *T. forsythia* (*Tf*QC), and *P. intermedia* (*Pi*QC), all bacteria that are strongly associated with periodontal disease (2). The crystal structures of *Pg*QC and *Tf*QC at 2.8 and 2.1 Å resolution clearly define them as type II QCs, with notable differences to their animal counterparts. These differences allow for the development of specific inhibitors of the bacteroidal enzymes, and we demonstrate with one such *Pg*QC inhibitor the successful inhibition of bacterial growth in a dose-dependent manner. Together, these data provide an excellent starting point for structure-based development of selective small-molecule inhibitors to target pathogenic oral microbes.

## Results and discussion

### Bacteroidal QCs exhibit analogous enzymatic characteristics to human QC

Kinetic measurements using the artificial fluorometric substrate H-Gln-AMC (43) clearly show that all three recombinant bacteroidal enzymes exhibit QC activity (Table 1; Fig. S1*A*). *Pg*QC and *Pi*QC exhibit an approximately tenfold and *Tf*QC a 20-fold higher K_M_ value compared with the human enzyme *Hs*QC (44). In contrast, the k_cat_ values of the bacteroidal proteins are in a similar range to that of *Hs*QC, although *Pi*QC possesses a twofold higher turnover number than *Pg*QC or *Tf*QC (Table 1). Consequently, the specificity constants k_cat_/K_M_ are approximately 4% (*Tf*QC), 9% (*Pg*QC) and 13% (*Pi*QC) that of the *Hs*QC for H-Gln-AMC. In each case, their pH activity profiles fit to classical bell-shaped curves (Fig. S1*B*). All bacteroidal QCs exhibit maximum activity in the mild alkaline range, with *Pg*QC possessing high enzymatic activity over a broader pH range (pK_a1_= 6.55 and pK_a2_ = 8.78) than *Pi*QC (pK_a1_ = 7.04 and pK_a2_ = 7.78) and *Tf*QC (pK_a1_ = 7.05 and pK_a2_ = 7.81).

**Table 1:** Kinetic parameters for the cyclization of H-Gln-AMC by bacteroidal QCs

Enzyme	K_M_ (μM)	k_cat_ (s^−1^)	k_cat_/K_M_ (mM^−1^ s^−1^)
*Pg*QC	510 ± 13	4.71 ± 0.22	9.24 ± 0.28
*Tf*QC	1090 ± 30	4.49 ± 0.22	4.1 ± 0.14
*Pi*QC	645 ± 7	8.51 ± 0.37	13.2 ± 0.1
*Hs*QC^a^	54 ± 2	5.3 ± 0.1	98 ± 2

a Data from Schilling *et al.* (44).

As described previously for *Hs*QC (44, 45), the pK_a1_ values indicate that an uncharged N terminus of the glutamine residue in H-Gln-AMC is necessary for bacteroidal QC activity, while the pK_a2_ values may reflect dissociation of a protonated group in the active center. All three bacteroidal type II QCs exhibit pH characteristics similar to those of *Hs*QC, which possesses an optimum at mildly alkaline pH with pK_a1_= 6.8 and pK_a2_ = 8.6 (43). As in gram-negative bacteria, the periplasmatic pH equilibrates with the environmental pH (46), *Pg*QC activity could be influenced by sulcular and salivary pH. Measurements of pH in the periodontal pocket have shown an increase to higher values up to pH 8.4 in severe inflammation (47, 48). This in turn is thought to promote the growth of more acid-sensitive bacteria such as *P. gingivalis* and *T. forsythia*. Recent studies have demonstrated an increased salivary pH of subjects with gingivitis or generalized chronical periodontitis compared with healthy controls, in line with the idea that alkaline pH is necessary for plaque growth (49, 50, 51). These observations are consistent with the growth pH optimum of *P. gingivalis* at pH 7.5 (52) and the optimal enzymatic activity of *Pg*QC as well as for *Tf*QC and *Pi*QC at alkaline pH.

### Bacteroidal QCs behave as human type II QC metalloenzymes

The crystal structure of human QC and additional enzymatic characterizations of numerous type II QCs indicate a metal-dependent catalytic mechanism (32, 45), although recent studies on tick QC from *Ixodes scapularis* (28) suggest that this might not be universal (53). Similar to the situation with *Hs*QC (45), EDTA has little influence on bacteroidal QCs, even at high concentrations (Fig. S2*A*). In contrast, dipicolinic acid and 1,10-phenanthroline (Figs. S2*B* and S3*C*) show strong inhibitory activity toward all three bacteroidal QCs, with 1,10-phenanthroline inhibiting QC activity effectively in the low mM range. *Pi*QC appears to be particularly sensitive to 1,10-phenanthroline. Thus, bacteroidal QC activity is inhibited by chelator-dependent metal depletion, in line with their classification as type II QC metalloenzymes.

The inhibitory effects of the heterocycles imidazole, benzimidazole, and 1-benzylimidazole were also investigated (Table 2). All three bacteroidal QCs were inhibited in a competitive manner, as demonstrated for *Pg*QC in the presence of 1-benzylimidazole (Fig. S3). The estimated Ki values are in the μM range, with 1-benzylimidazol showing the highest efficacy with about 10 μM. Interestingly, benzimidazole is more reactive against bacteroidal QCs than imidazole (Table 2), which is contrary to the situation for human QC (43), suggesting a different chemical environment in their active sites.

**Table 2:** Inhibition of QC (K_i_, μM) catalyzed reaction in the presence of different heterocycles

Heterocycle	*Pg*QC	*Tf*QC	*Pi*QC	*Hs*QC
Imidazole	349 ± 23	280 ± 15	288 ± 11	222 ± 21
Benzimidazole	32.1 ± 1.4	170 ± 1	85.5 ± 4.7	318 ± 23
1-Benzylimidazol	10.4 ± 0.3	6.45 ± 0.33	13.5 ± 2.2	16.8 ± 3.5

### Structures of *Pg*QC and *Tf*QC reveal characteristics of bacteroidal type II QCs

**Figure 2:**
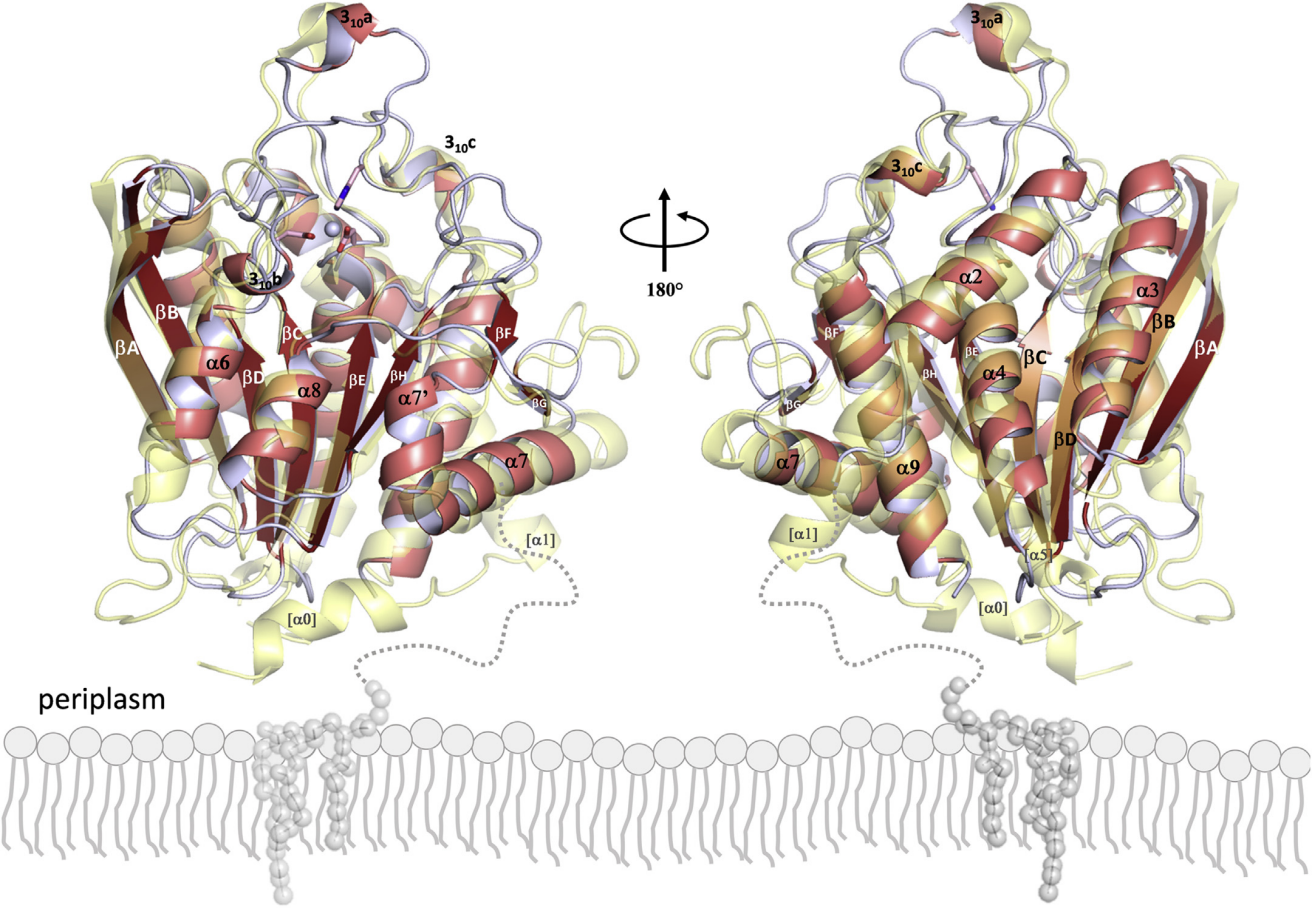
**Front (left) and back (right) views of the overall structure of *Pg*QC (cartoon representation, *red tones*) overlaid with that of *Hs*QC (*transparent yellow*).** The active site zinc atom is shown as a *gray sphere*, with ligating residues Asp149, Asp183, and His299 shown in stick representation. The putative membrane anchor (an N-acetylated Cys21-linked diacylglycerol moiety) is depicted schematically as *gray balls* and *sticks*, embedded in the outer leaflet of the bacteroidal cytoplasmic membrane.

*Pg*QC crystallized in a trigonal crystal form with two monomers in the asymmetric unit. Both monomers display a compact architecture of the type II QC α/β-hydrolase fold (54): an open-sandwich topology with a twisted central six-stranded β-sheet βA–βB–βC–βD–βE–βH (with βB running antiparallel to the others), surrounded on the concave side by helices α6 and α8, on the convex side by α2, α3, α4, and α9, and closed on one edge by the segment βF–α7’–α7–βG (Fig. 2, Fig. S4). Although the crystallized construct includes residues corresponding to Asn21-Gln40, *Pg*QC lacks the first two helices α0 and α1 present in *Hs*QC, with density defined for residues Ala41–Lys329 only. Several of the loops connecting secondary structure elements are shorter compared with *Hs*QC, as are helices α6 and α7, and helix α5 is missing altogether. In contrast, helix α7 of *Pg*QC, which is shifted by half a pitch compared with its *Hs*QC counterpart, is preceded by the short helix α7’. *Pg*QC also presents three short 3_10_-helices in the loops between βC and α4 (3_10_a), βD and α6 (3_10_b), and βH and α9 (3_10_c), the latter also a feature of *Hs*QC (Fig. 2; Fig. S4).

**Figure 3:**
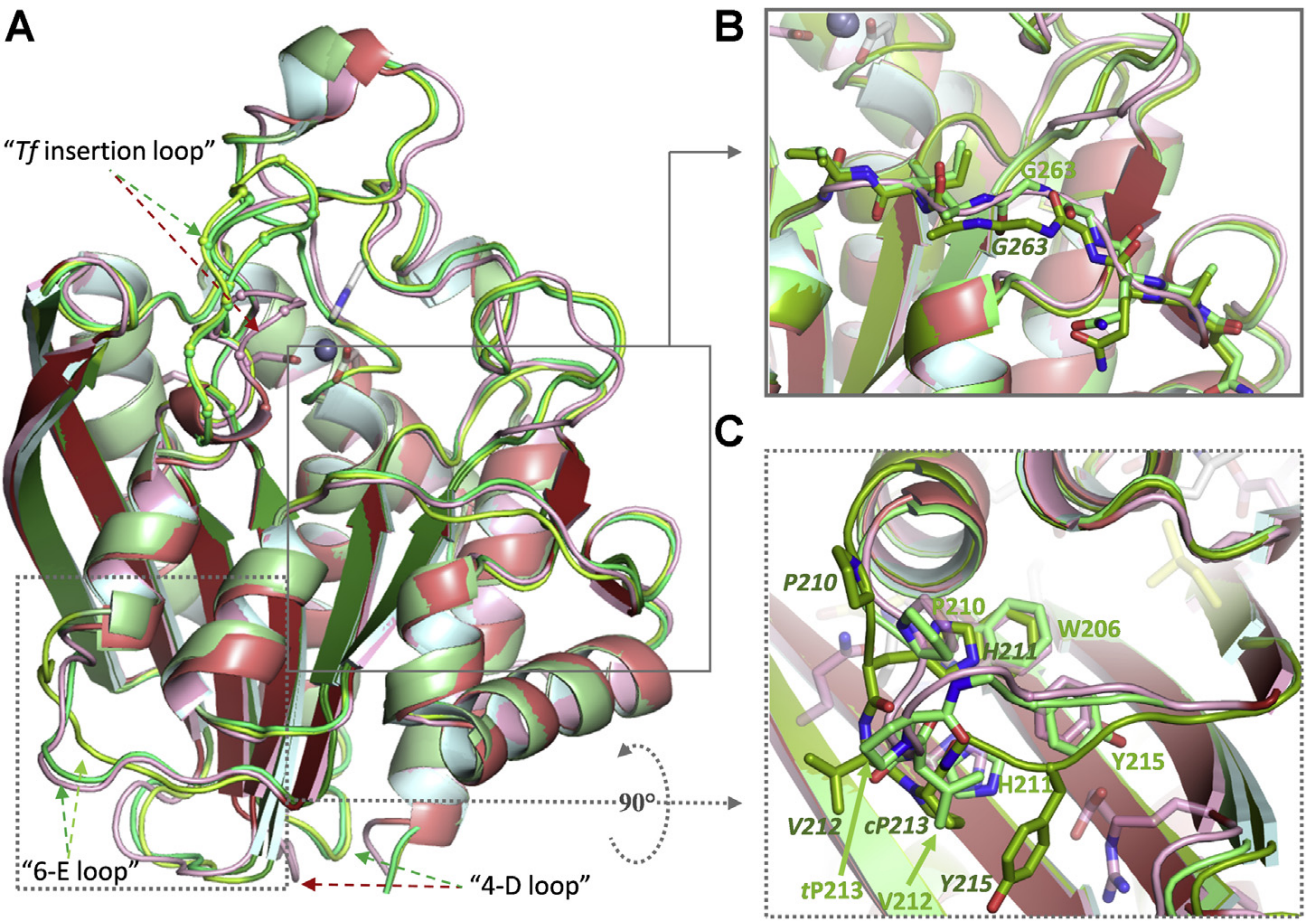
**Superposition of the crystal structures of *Tf*QC (*green tones*) reveals the same overall architecture (*A*) as *Pg*QC (*red tones*, front orientation as defined in**Fig. 2**).** Main chain differences are observed in the “*Tf* insertion” and “4-D” loops. The four monomers in the *Tf* crystal asymmetric unit reveal two separate conformations demonstrated for monomers A (*dark green*, shared with C) and B (*apple green*, shared with D). *B*, structural plasticity at the entrance to the active site in mouse QC (*Mm*QC) (55) is also observed for the two *Tf*QC conformations. *C*, due to *cis*-/*trans*-isomerization of the *Tf*Val212-Pro213 peptide bond, two routes are observed for the “6-E” loop in *Tf*QC, resulting in a slight reorientation of the α6 helix (see Movie S1). Residues conserved in *Pg*QC-like sequences (Fig. S4, Movie S2) are shown as *pink sticks*.

The same architecture is observed for *Tf*QC, with an r.m.sd to *Pg*QC of 1.1 Å for 286 Cα atoms (Fig. 3). The only significant differences are seen in an altered course of the loop between α4 and βD (“4-D loop”, presumably due to the presence of *Tf*Pro171) and a lengthened loop connecting βD and α6 (“*Tf* insertion loop”, which starts with conserved residues *Pg*Asp183 and ends with *Pg*Trp193). A small degree of structural variation is observed between two sets of crystallographically independent *Tf*QC monomers in the loop containing *Tf*Gly268-Gly269 that connects βG and α8 (Fig. 3*B*), which was also observed for mouse QC (*Mm*QC) (55). Interestingly, two distinct conformations of the loop connecting α6 and βE (“6-E loop”) were observed in *Tf*QC: the *Tf*Val212–Pro213 peptide bond adopts a *cis*-conformation in two of the monomers as opposed to the *trans*-conformation found in *Pg*QC, leading to an alternative orientation of α6 (Fig. 3*C*; Movie S1). As a proline residue is found in this position only in *Tf*QC, this may be peculiar to the *Tannerella* enzyme, although it might also reflect an underlying plasticity of this region, which juxtaposes residues that are conserved in bacteroidal QCs (Gln90, Arg118, Asp175, Trp200, Pro204, His205, and Tyr209, *Pg*QC numbering; see [Fig. S4, Movie S2]).

**Figure 4:**
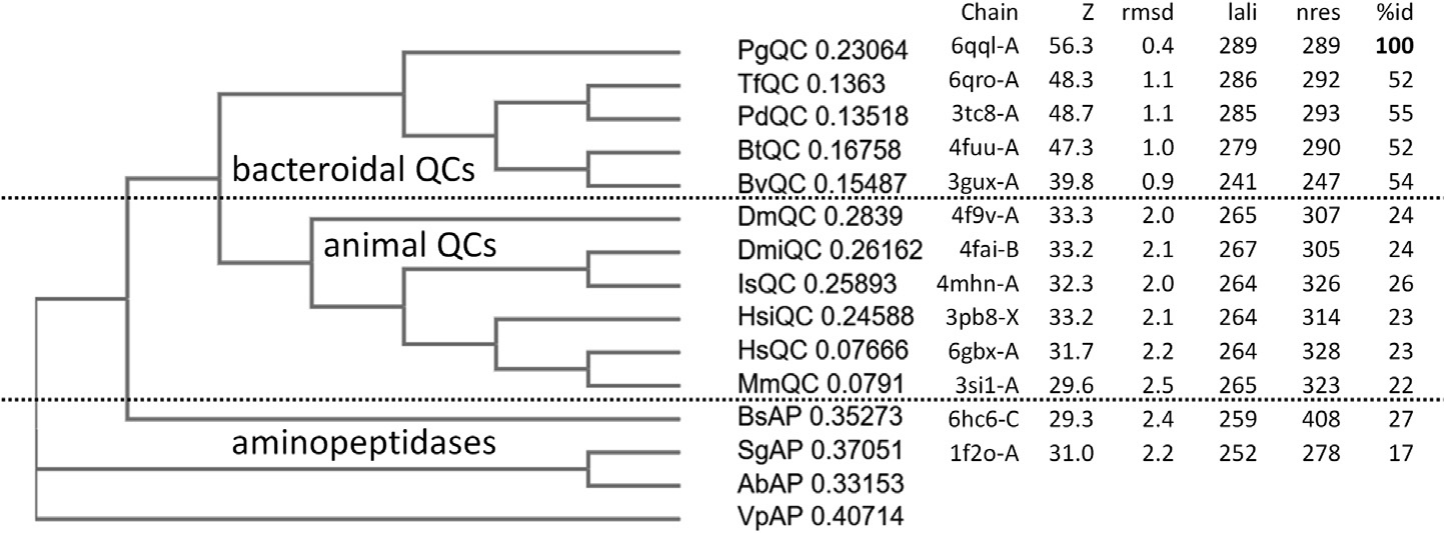
**Structural conservation between bacteroidal QCs, animal QCs, and aminopeptidases**. Phylogram based upon structure-based sequence alignment using the DALI server (44) (Fig. S4) using *Pg*QC monomer A as search model. QC sequences are from the organisms *Parabacteroides distasonis* (PdQC), *Bacteroides thetaiotaomicron* (BtQC), *Bacteroides vulgatus* (BvQC), *Drosophila melanogaster* (cytosolic DmQC and mitochondrial DmiQC), *Ixodes scapularis* (IsQC), *Homo sapiens* (Golgi-resident isoQC HsiQC and cytosolic HsQC), and *Mus musculus* (MmQC), and aminopeptidase sequences from *Bacillus subtilis* (BsAP) and *Streptomyces griseus* (SgAP). Aminopeptidases from *Aneurinibacillus sp. AM-1* (AbAP, 2ek8) and *Vibrio proteolyticus* (VpAP, 3b3s) were used as outgroup. Chain: pdb code and chain label; Z: DALI Z-score for coordinate sets; “rmsd”: root mean square deviation; lali: number of aligned residue pairs; nres: number of residues; %id: % sequence identity.

In addition to animal QCs, a search for similar structures in the PDB using DALI (56) retrieved three probable bacteroidal QC enzymes: bdi_3547 from *Parabacteroides distasonis* ATCC 8503, bt_2548 from *Bacteroides thetaiotaomicron* VPI-5482, and bvu_1317 from *Bacteroides vulgatus* ATCC 8482 (pdb codes 3tc8, 4fuu and 3gux respectively, unpublished) (Fig. 4). All three structures, solved as part of the human gut microbiome project at the Joint Center for Structural Genomics (57), have been annotated as putative Zn^2+^-dependent aminopeptidases (APs). The structural similarity to *Pg*QC and *Tf*QC is striking (Fig. S5*A*), with more than 50% sequence identity to *Pg*QC, conservation of all residues conserved in *Pg*QC-like sequences (Fig. S4, Movie S2), and only minor differences in loop size and orientation. In contrast to the APs (that possess a double-zinc active site (58) and to which they display 25–30% sequence identity) but in common with *Pg*QC and *Tf*QC, only one zinc ion is found in both 3tc8 and 4fuu, coordinated in *Pg*QC by *Pg*Asp149, *Pg*His299, and (bacteroidal QC-specific) *Pg*Asp183.

Loss of the second zinc ion binding site, which allows accommodation and protonation of the substrate glutamine Nε2 leaving group (59), is most likely linked to the presence of a *trans*-peptide bond between *Pg*Asp149 and *Pg*Gly150, resulting in an alternative route of the main chain (Fig. S5*B*). In the aminopeptidase from *Streptomyces griseus* (*Sg*AP), the equivalent *Sg*AP–Asp97–Asn98 peptide bond is in *cis*-conformation, with the *Sg*AP–Asn98 side chain making hydrogen bonds to the main chain carbonyl oxygen of *Sg*AP–Phe159 and backbone amides of *Sg*AP–Gly101 and *Sg*AP–Met161. Interestingly, the residue corresponding to *Sg*AP–Asn98 is consistently an asparagine or aspartate in APs (Fig. S4), whereas a serine residue (*Hs*QCSer160) with preceding *cis*-peptide bond occupies the corresponding position in animal QCs. It is noteworthy that *Pg*QC shares the presence of *Pg*Trp298/*Pg*Phe294 with animal QCs, which are Tyr/a small aliphatic residue in APs, respectively. Based on the strong similarities in primary and tertiary structure, we propose that bdi_3547 (3tc8) and bt_2548 (4fuu) be reannotated as *Pd*QC and *Bt*QC respectively. Surprisingly, no density for a zinc ion is found for bvu_1317 (3gux, which we denote as *Bvu*QC), despite an amino acid sequence that bears all the hallmarks of a bacteroidal QC (Fig. S4, Movie S2). Indeed, this is accompanied by a lack of density for four surface loops that surround the active site (Fig. S6), suggesting that zinc removal might lead to partial unfolding of *Bvu*QC and its inactivation.

### Access to the active site is restricted in bacteroidal QCs

**Figure 5:**
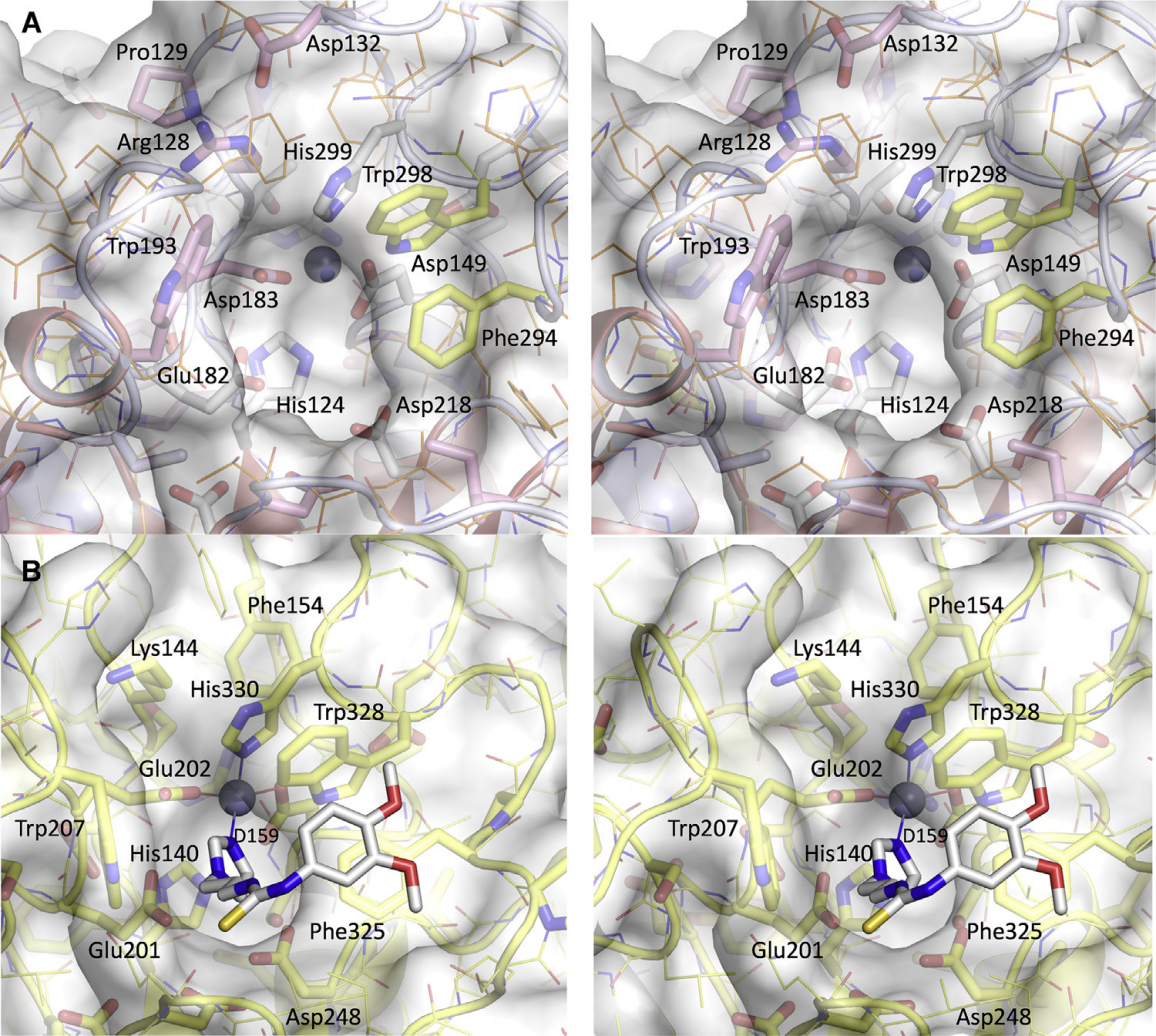
**Access to the active site of *Pg*QC (*A*) is restricted compared with that of *Hs*QC (*B*) (wall-eyed stereo; orientation is rotated 90° about a horizontal axis compared with the front orientation)**. *Pg*QC residues conserved in bacteroidal QCs, glutaminyl cyclases, and aminopeptidases are shown in *pink*, *yellow*, and *white*, respectively (see also Fig. S4, Movie S2). To aid comparison, the *Hs*QC inhibitor PBD150 (pdb code 3pbb) is shown as *white sticks*.

The active site and substrate binding regions are located at the C-terminal edge of the central QC β-sheet. As noted above, the single-zinc active site of *Pg*QC bears a strong resemblance to that of animal QCs (Fig. 5). Replacement of the third zinc ligand (*Hs*Glu202) with the shorter *Pg*Asp183 results in a minor rearrangement of the main chain. Of note, the indole ring of *Pg*Trp193 (conserved in bacteroidal QCs, [Fig. S4, Movie S2]), which is C terminal to the “insertion loop” and prior to α6, occupies a similar spatial position to that of *Hs*Trp207, which forms one edge of the active site pocket and has been shown to participate in substrate binding and catalysis (32). As a result of a different main chain position of *Pg*Trp193 and resulting side chain orientation, the active site is much deeper than in animal QCs. Access is further restricted by (i) the loop following strand βC formed by residues *Pg*Arg128, *Pg*Pro129, and *Pg*Asp132 (all conserved in bacteroidal QCs), (ii) the inserted loop following βH immediately prior to *Pg*Phe294 and *Pg*Trp298 (both conserved in all type II QCs and that presumably play a role in substrate binding [55]), as well as (iii) the extended chain between βG and α8 that shows structural variability in *Tf*QC (see Fig. 5) and in mammalian QCs (55). These differences in active site architecture promise to facilitate the search for inhibitors specific to bacteroidal QCs.

### Modification of potential physiological substrates by *Pg*QC

In comparison to other bacterial phyla, species belonging to the *Bacteroidetes* phylum possesses disproportionally high numbers (up to 80%) of genes encoding proteins with a signal peptide followed by Gln that are potential substrates for QC (21). In *P. gingivalis* outer membrane vesicles (OMVs) alone, 24 proteins with potential N-terminal pGlu residues were detected, most of them secreted *via* T9SS. Although cargo proteins of T9SS are essential for *P. gingivalis* virulence, they are dispensable for the bacterium growth *in vivo* (60), so that it is likely that pyroglutamination is important for integrity and/or function of essential proteins exported to the periplasm or into the outer membrane. Indeed, several essential genes identified in the *P. gingivalis* genome (41) encode proteins containing an N-terminal signal peptide with a Xaa-Gln cleavage motif. We selected five putative *P. gingivalis* substrates: N-acetyl-muramoyl-L-alanine amidase (PG_0076), a putative PorT family protein (PG_0083), a conserved hypothetical protein (PG_0140), a putative OM-β-barrel protein (PG_0234), and a predicted TolC protein (PG_0285). All five synthesized Gln_1_-Xaa_2-10_ decapeptides are *in vitro* substrates for *Pg*QC (Table 3), with specificity constants k_cat_/K_M_ comparable with that of the H-Gln-AMC substrate. For *Hs*QC (44), extended peptide substrates show up to 20-fold higher specificity constants compared with H-Gln-AMC, although this can vary greatly. The peptide corresponding to N-acetylmuramoyl-L-alanine amidase shows an approximately fivefold increase in k_cat_/K_M_ to 65 mM^-1^s^-1^. The latter enzyme, which belongs to the class of peptidoglycan amidohydrolases, has been implicated in the biogenesis of OMVs and in the establishment of pathogenic biofilms through cell wall hydrolysis of resident bacteria of the healthy biofilm (61, 62, 63, 64).

**Table 3:** Kinetic parameters for the conversion of N-termini of putative natural substrates by *Pg*QC

Predicted substrate	Synthetic peptide	K_M_ (μM)	k_cat_ (s^−1^)	k_cat_/K_M_ (mM^−1^ s^−1^)
N-acetylmuramoyl-L-alanine amidase	QSRNRTYEAY	203.7 ± 5.1	13.3 ± 1.6	65.1 ± 6
PorT family protein	QESNASVRPS	n.d.	n.d.	4.3 ± 0.1^a^
Conserved hypothetical protein	QSVVPDSIGR	492.5 ± 26	7.0 ± 0.66	14.3 ± 0.6
Immunoreactive 23 KDa antigen	QDVIRPWSLQ	n.d.	n.d.	18.6 ± 0.1^a^
TolC family protein	QQVAAADPSP	n.d.	n.d.	12.5 ± 1.9^a^

a determined under first-order-rate law conditions, *i.e.*, at [S] << K_M_.

### Bacterial growth attenuation by *Pg*QC inhibition

**Figure 6:**
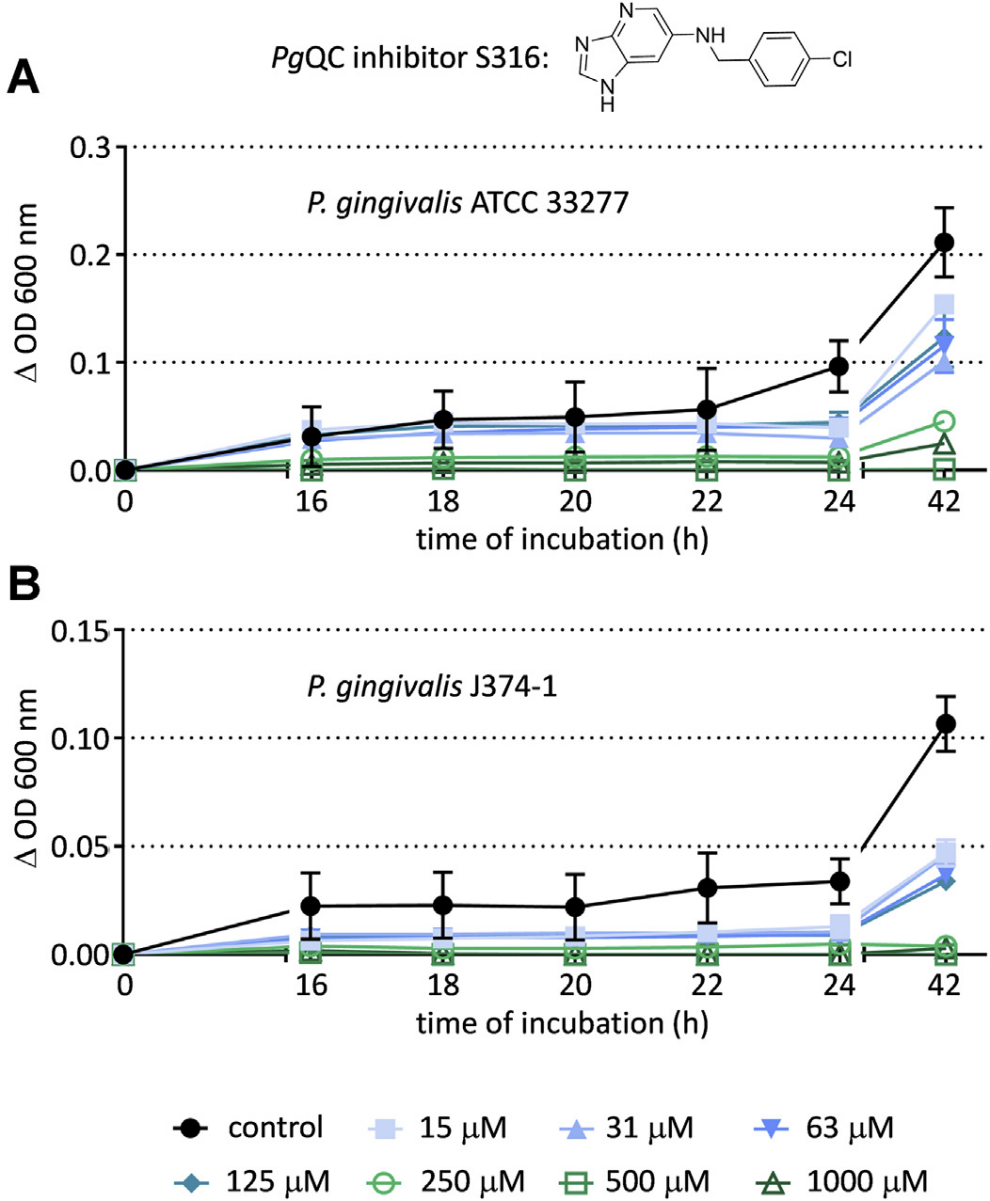
***Pg*QC-specific inhibitor S-316 suppresses *P. gingivalis* growth in a concentration-dependent manner**. Growth curves of *P. gingivalis* ATCC 33277 (*A*) and *P. gingivalis* J374-1 (*B*) in the presence and absence of varying concentrations of the inhibitor. Standard deviations from the 96-well plates (independent replicates) are shown.

Saturation transposon insertion sequencing of *P. gingivalis* has identified *Pg*QC as a candidate essential gene (41, 42); indeed, all our attempts to generate a *Pg*QC knockout strain have failed until now (unpublished results). An ongoing medicinal chemistry program involving extensive modeling and synthesis was initiated to generate inhibitors for *Pg*QC. One of the first compounds to exhibit significant effects is compound S-316 (*N*-(4-chlorobenzyl)imidazo[4,5-b]pyridin-6-amine), with a K_i_ value of 53 ± 1.1 nM for *Pg*QC and 655 ± 27 nM for *Hs*QC. The compound was used as a chemical probe for target validation in bacteria culture using the micro-broth dilution technique, an accepted procedure in testing activity of antimicrobials against slowly growing bacteria such as anaerobic bacteria. Minimal inhibitor concentrations (MICs) for *P. gingivalis* (a laboratory strain as well as two clinical isolates), *T. forsythia*, and *P. intermedia* were each determined to be submillimolar (Table 4). On the other hand, application to the Gram-positive *Streptococcus gordonii* and Gram-negative *Fusobacterium nucleatum*, which do not possess a QC-like enzyme, did not result in growth attenuation. *P. gingivalis* growth inhibition was dose-dependent for both the laboratory strain and a clinical isolate (Fig. 6).

**Table 4:** Bacterial growth attenuation by *Pg*QC inhibitor S-316

Species	*Porpyhromonas gingivalis*			*Tannerella forsythia*	*Prevotella intermedia*	*Streptococcus gordonii*	*Fusobacterium nucleatum*
Strain	ATCC 33277	M5-1-2^a^	J374–1^a^	ATCC 43037	ATCC 25611	ATCC 10558	ATCC 25586
MIC (μM)	500	250	500	500	125	>2000	>2000

No differences were observed between individual replicates.

a Clinical isolates.

In summary, we have demonstrated glutaminyl cyclase activity from heterologously produced recombinant enzymes from the oral pathogens *P. gingivalis*, *T. forsythia*, and *P. intermedia* and that *Pg*QC is able to convert peptide substrates representing the mature N-termini of *P. gingivalis* proteins. Crystal structures reveal their structural similarity to eukaryotic type II QCs, including human QC, and thus represent the first examples of prokaryotic Type II QCs. Although the active centers of all type II QCs are highly similar, enabling similar inhibition mechanisms *via* zinc chelators, selectivity for bacteroidal type II QC inhibition is possible through differences in the substrate binding pockets. Indeed, the selective *Pg*QC inhibitor S-316 shows efficacy in inhibiting growth of each of the aforementioned oral pathogens, indicating that the bacteroidal QCs may be essential for cell viability. Although these data are very promising, further *in vitro* and *in vivo* studies are necessary to determine the influence of N-terminal pyroglutamate modification on substrate protein stability and functionality and to determine the relationship between enzyme inhibition and bacteriostasis. Nevertheless, the results presented here demonstrate that bacteroidal QCs are a promising target for drug development in the treatment of periodontitis and associated diseases including AD and rheumatoid arthritis.

## Experimental procedures

### Molecular cloning procedures

*Escherichia coli* strain DH5α (Invitrogen) or XL-1 blue (Stratagene) was used for cloning procedures. *E. coli* Rosetta(DE3)pLysS (Novagene) was used for protein expression. All *E. coli* strains were grown in Luria–Bertani medium as indicated at 20 °C, 30 °C, or 37 °C. Antibiotics (ampicillin [50–125 mg/l], chloramphenicol [15–30 mg/l], and kanamycin [25 mg/l]), IPTG, and ethanol were added where appropriate. For preparation of solid media, 1.5 % agar (Roth) was added to corresponding broth.

All cloning procedures were performed applying standard molecular biology techniques. For protein expression, ORFs of *Pg*QC [QC from *P. gingivalis*, KEGG Genome PG_2157], *Tf*QC [QC from *T. forsythia*, BFO_1693], and *Pi*QC [QC from *P. intermedia*, PIN17_A0594]) were amplified using the synthesized DNA sequence, purchased from Eurofins Genomics, as template in a PCR to introduce a *Nhe*I/*Nde*I restrictions site for direct cloning into the vector pET28a(+) (Novagen). Putative signal sequences were replaced by N-terminal His-tags.

### Protein expression

The expression vectors pET28a(+)::*Pg*QC, pET28a(+)::*Pi*QC and pET28a(+)::*Tf*QC were each transformed in *E. coli* Rosetta(DE3)pLysS. Bacteria were grown in Luria–Bertani medium containing kanamycin (25 μg/ml) and chloramphenicol (15 μg/ml) at 37 °C overnight. Overnight culture was diluted 1:100 in fresh Luria–Bertani medium and incubated at 37 °C until the cell density reached an OD_600_ ∼0.6. The cultures were induced with 0.4 mM isopropyl β-D-1-thiogalactopyranoside (IPTG) and a 2% (v/v) ethanol volume was added, followed by an incubation time for 16 h at 20 °C with continuous shaking. Cultures were harvested by centrifugation at 4 °C and 3900*g* for 30 min and cell pellets were stored at –20°.

### Protein purification

Cell pellets of 500 ml cultures were resuspended in 20 ml buffer consisting of 50 mM Tris-HCl, 150 mM NaCl pH 8.0, 10 μg/ml DNase, 1 mM MgCl_2_, and protease inhibitor cocktail mix (complete mini tablets, EDTA-free, Roche). Cells were disrupted by passing through a French Press (Thermo Scientific) for 3–4 times. Cell debris was removed by centrifugation at 4 °C and 30,000*g* for 30 min. Supernatant was loaded on a 5 ml HisTrap column (GE Healthcare) or in the case of *Tf*QC on a 5 ml HiTrapTalon column (GE Healthcare). The column was equilibrated with 50 mM Tris-HCl pH 8.0 containing 150 mM NaCl. Unbound fractions were removed by washing with several column volumes of equilibration buffer containing 20 mM imidazole. Proteins were eluted using multiple step gradients, reaching a final concentration of 250 mM imidazole, with the majority of pure protein eluting at approximately 100 mM imidazole. All QC-containing fractions were pooled, concentrated with Vivaspin 20 (Sartorius AG), and applied to a HiPrep 26/10 desalting column (GE, Healthcare) equilibrated with 50 mM Tris-HCl, 150 mM NaCl, pH 8.0. The purification was analyzed by SDS-PAGE and the protein content was determined by absorption at 280 nm using a NanoDrop 2000 spectrophotometer (Thermo Scientific) or according to the methods of Bradford or Gill and von Hippel (65, 66). Yields of up to 40 mg recombinant His-tag fusion proteins were obtained from ca. 500 ml induced *E. coli* cultures. Finally, purified recombinant QC proteins were shock-frozen in liquid nitrogen and stored at –80 °C.

N-terminal His-tags of the fusion proteins were removed using 1 unit of biotinylated thrombin per 1 mg fusion protein (Thrombin cleavage Capture Kit, Novagen) in the presence of 20 mM Tris-HCl, 150 mM NaCl, 2.5 mM CaCl_2_ at pH 8.0 for 16 h at 4 °C. Thrombin was eliminated through binding to Streptavidin Agarose (Thrombin cleavage Capture Kit, Novagen) and QC proteins without His-tag were recovered by spin filtration. The purified proteins therefore possess the N-terminal sequence Gly-Ser-His-Met from the vector, followed by *Pg*Asn21 (*Pg*QC) or *Tf*Gly22 from the bacteroidal QC sequences. After further desalting step using HiPrep (26/10), QC fractions were pooled and concentrated with Vivaspin 20 (Sartorius AG). Recombinant purified QCs (Fig. S7) in 50 mM Tris-HCl, 150 mM NaCl, pH 8.0 were shock-frozen in liquid nitrogen prior to enzymatic characterization and stored at –80 °C.

### Determination of QC activity

QC activity was evaluated using the fluorogenic substrate H-Gln-AMC as described previously (43). Measurements were performed at different concentrations of fluorogenic substrate and 0.5 U pyroglutaminyl aminopeptidase in 50 mM Tris-HCl, 50 mM NaCl at pH 8.0. After 10 min incubation time at 30 °C, the reaction was initiated by addition of approximately 50 nM QC to a final volume of 125 μl reaction mixture. QC activity was calculated from a standard curve of the fluorophore AMC at assay conditions using excitation/emission wavelengths of 380/460 nm. All measurements were carried out in 96-well microtiter plates (Fisher Scientific) at 30 °C using the FluoStar Optima (BMG Labtech). All kinetic data were evaluated using GraFit software (Version 7, Erithacus software Ltd).

For investigation of pH dependency of QC activity, a three-component buffer consisting of 0.05 M acetic acid, 0.05 M MES, 0.1 M Tris, and 0.05 M NaCl at a pH range from 6.0 to 8.5 was used. This buffer provides a constant ionic strength over a broad pH range. All QC-activity determinations were carried out under first-order rate law conditions, *i.e.*, at substrate concentrations at 1/10 K_M_. Thus, the results represent the pH dependence of the specificity constant k_cat_/K_M_. Because of the reduced stability of the auxiliary enzyme pyroglutamyl aminopeptidase under acid or basic conditions, pH dependency of QCs cannot be determined below pH 6 or above pH 9. The resulting kinetic data were fitted empirically with a two-residue ionization mechanism described by a “bell-shaped” curve.

To test whether QC is a metalloenzyme, the turnover of H-Gln-AMC at a concentration corresponding to 0.5 K_M_ was investigated in the presence of the metal chelators 1,10- phenanthroline, dipicolinic acid, and EDTA. The fluorometric activity assay was performed as described above, except for the presence of 1% (v/v) DMSO in the case of 1,10-phenanthroline or dipicolinic acid. Finally, the residual QC activity was determined at steady state, whereas QC without chelators and ± 1% DMSO served as positive control.

For inhibitor testing, heterocycles imidazole, benzimidazol, and 1-benzylimidazol were added with 1% (v/v) DMSO in the reaction mixture. Inhibitory constants were determined using concentrations of H-Gln-AMC varying from 1/4 K_M_ to 2 K_M_ and a final concentration of bacterial QC in a range between 25 nM and 50 nM. Progress curves were fitted to the general equation for competitive inhibition.

For evaluation of possible physiological substrates, *Pg*QC activity was determined spectrophotometrically by using glutamate dehydrogenase as auxiliary enzyme described previously (44). The assay consists of varying concentration of synthetic substrate, 0.3 mM NADH, 13 mM α-ketoglutaric acid, and 3.25 U glutamic dehydrogenase in 50 mM Tris-HCl, 50 mM NaCl at pH 8.0. After 10-min incubation time at 30 °C, reactions were initiated by addition of approximately 115 nM *Pg*QC to a final volume of 250 μl reaction mixture. QC turnover of substrates was monitored by a decrease of absorption at 340 nm and enzyme activity was determined from a standard curve of NH_4_Cl at assay conditions. All determinations were carried out in 96-well microtiter plates (Fisher Scientific) using Sunrise (Tecan).

### Protein crystallization

Bacteroidal QCs were crystallized using the hanging as well sitting drop vapor diffusion technique at 15 °C. For *Pg*QC, 1 μl of a 90 μM protein solution (3.2 mg/ml in 50 mM Tris-HCl pH 8.0, 150 mM NaCl) was mixed 1:1 with reservoir buffer and placed over 500 μl crystallization buffer in EasyXtal 24-well plates (Qiagen) using the hanging drop technique. Crystals appeared within 40 days in 0.1 M HEPES pH 8.0, 3 % (v/v) PEG 400, and 2 M (NH_4_)_2_SO_4_. Prior to X-ray analysis, crystals were cryoprotected by rapid soaking in crystallization buffer containing 18 % (v/v) glycerol and subsequently flash-frozen at –180 °C using an X-Stream cryo-nitrogen stream (Rigaku/MSC). For *Tf*QC, 200 nl of a protein solution (279 μM; 9.91 mg/ml in 50 mM Tris-HCl pH 8.0, 150 mM NaCl) was mixed 1:1 with crystallization buffer, consisting of 2 M (NH_4_)_2_SO_4_ and 0.1 M sodium acetate pH 4.6 (JBScreen Classic 6), and sealed over 55 μl reservoir solution in SWISSCI MRC 3 Well Crystallization Plate (UVP) using a Cartesian pipetting robot. The crystallization process was followed using a crystallization plate-imaging system (Desktop Minstrel UV; Rigaku Europe), with crystals appearing after 3 months. Crystals were cryoprotected by soaking in mother liquor containing 25 % (v/v) ethylene glycol and flash-frozen at –180 °C prior measurement.

### Structure solution and refinement

Data sets were collected in-house using a CCD detector (SATURN 944+, Rigaku Europe) mounted on a copper rotating-anode source (RA Micro 007, Rigaku Europe). Diffraction data were integrated, scaled, and merged using XDS (67). *Pg*QC crystals belong to the trigonal space group P3_1_21 with cell constants a= 89.9 Å, b= 89.9 Å, c= 164.7 Å, α = 90°, β = 90°, γ = 120° and diffract to a resolution of 2.8 Å. Initial phases for the *Pg*QC structure were obtained by molecular replacement using the program PHASER MR from the CCP4 crystallographic suite with chain A of the human QC structure (PDB code 2AFM) as search model (68). Initial automatic model building and refinement using PHENIX.AUTOBUILD were followed by several cycles of manual rebuilding using the program COOT and maximum-likelihood refinement using PHENIX.REFINE with NCS restraints (69). The final model could be refined to R_work_/R_free_ values of 0.22/0.26 and contains two molecules in the asymmetric unit, comprising residues A^41^-K^329^ for chain A and A^41^-V^328^ for chain B. The lack of density for the first 20 residues is in keeping with a high intrinsic disorder prediction of the sequence ^21^NGNNTSETQGDRTEQAETVQ^40^ (PrDOS server http://prdos.hgc.jp/).

Initial phases for the *Tf*QC crystal, which diffracted to a resolution of 2.1 Å, were obtained by molecular replacement using PHASER MR from the CCP4 crystallographic suite. Again, chain A of human QC (PDB code 2AFM) was used as search model, but was modified by side chain pruning using the program SCULPTOR from the PHENIX suite. Although initially indexed in a monoclinic space group, further analysis revealed the crystal to belong to the triclinic space group P1 (cell constants a= 56.1 Å, b= 79.2 Å, c= 83.1 Å, α = 89.9°, β = 90°, γ = 71.9) with pseudo-merohedral twinning according to X-TRIAGE (twin law -h, -k, l) and four molecules in the asymmetric unit. The final *Tf*QC structure was obtained after repeated cycles of manual rebuilding using the program COOT and maximum-likelihood refinement incorporating twin (amplitude) refinement using PHENIX.REFINE. The final model could be refined to R_work_/R_free_ values of 0.17/0.20 and contains residues P^44^ to K^334^ in all four chains. As with *Pg*QC, the missing sequence ^22^GQKNTTKEETTEPADTDKRIE^42^ is likely to be disordered. Data collection and refinement statistics are given in Table 5.

**Table 5:** Statistics for data collection and refinement. (Statistics for the highest-resolution shell are shown in parentheses)

Crystallographic statistics	*P. gingivalis* QC	*T. forsythia* QC
Data collection statistics		
Radiation source	Rotating anode	Rotating anode
Wavelength (Å)	1.5418	1.5418
Space group	P3_1_21	P1
Unit cell length (Å)	89.6, 89.6, 164.2	56.1, 79.2, 83.1
Unit cell angles (°)	90, 90, 120	89.9, 90.0, 71.9
Resolution range (Å)	44.9–2.81	38.3–2.10
Highest resolution shell (Å)	2.97–2.81	2.18–2.10
R_meas_	38.7 (134.0)	14.7 (58.2)
I/σI	5.35 (1.33)	10.15 (2.44)
Completeness (%)	99.6 (98.3)	96.0 (73.9)
CC (1/2)	96.4 (55.5)	98.9 (63.6)
Multiplicity	5.6 (5.5)	3.6 (2.6)
Solvent content (%)/QC per ASU	55/2	50/4
Wilson B factor	40.7	17.4
Refinement statistics		
Number of reflections (working/test set)	19,348/970	76,199/3810
R_work_/R_free_	0.226/0.255	0.174/0.201
No. atoms		
Protein	4475	9240
Ligand	7	69
Water	23	599
Average B-factors (Å^2^)	40.5	16.6
Protein	40.5	16.6
Ligand	76.6	22.7
Water	29.3	15.7
Bond length r.m.s.d. (Å)	0.007	0.007
Bond angles r.m.s.d. (°)	0.83	1.01
Ramachandran plot (%): favored/outliers/allowed	97.6/0/2.4	98.6/0/1.4
MolProbity clashscore	2.99	8.80
PDB accession	6QQL	6QRO

R_meas_, redundancy independent indicator of data quality.

### Synthesis of potential peptide substrates

The synthesis of N-terminal peptides of potential native substrates was performed according to standard Fmoc-solid phase peptide synthesis on a Tetras peptide synthesizer (Advanced ChemTech). The peptides were synthesized at 60 μmol-scale as C-terminal amides on Rink amide resin (Merck Millipore) using standard Fmoc/tBu-protected amino acids. Coupling was performed with O-(benzotriazol-1-yl)-N,N,N,N-tetramethyluronium tetrafluoroborate (TBTU) and N-methylmorpholine (NMM). Fmoc-deprotection was carried out using 20% piperidine in DMF. Final cleavage and deprotection of the peptides were performed using TFA:EDT:TIS:H2O (50:2:1:1 v/v). After precipitation with cold diethylether, the crude peptides were collected by filtration and purified by semipreparative RP-HPLC. The purity and identity of the substrates were characterized by analytical RP-HPLC, MALDI-TOF MS, and ESI MS.

### Synthesis of *Pg*QC inhibitor S-316

**Figure 7:**
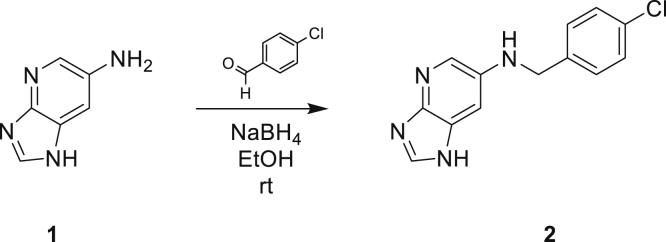
**Synthesis of S-316: *N*-(4-Chlorobenzyl)imidazo[4,5-b]pyridin-6-amine (2)**.

Imidazo[4,5-*b*]pyridin-6-amine (Fig. 7, [70]) (1, 50 mg, 0.4 mmol, 1 eq) and 4-chlorobenzaldehyde (52 mg, 0.4 mmol, 1 eq) were dissolved in EtOH (5 ml) and stirred at room temperature for 4 h. NaBH_4_ (21 mg, 0.6 mmol, 1.5 eq) was added and stirring was continued overnight. The reaction was quenched by means of water and extracted with EtOAc (3 × 20 ml). The combined organic layers were washed with brine, dried over Na_2_SO_4_, and evaporated. The crude product was purified by flash chromatography (silica, CHCl_3_/MeOH gradient). Yield: 48 mg (50%); MS m/z: 259.1 [M + H]^+^; HPLC: rt 9.97 min, 100%; ^1^H-NMR (DMSO-d6) δ: 4.33 (s, 2H); 6.39 (br s, 1H); 6.91 (s, 1H); 7.36 to 7.45 (m, 4H); 7.93 (d, 1H, ^4^J = 2.5 Hz); 8.08 (s, 1H); 12.22 (br s, 1H).

### Determination of minimal inhibitory concentration (MIC)

The micro-broth dilution technique was used to determine MICs. After subcultivation of bacterial strains and checking of purity, a defined inoculum was added to Wilkins–Chalgren broth (Oxoid) supplemented with 10 μg/ml β-NAD (Sigma-Aldrich) containing the inhibitor. A twofold dilution series was made, starting from 2 mM (final concentration) of the inhibitor. The growth control contained 10% of the inhibitor dissolvent at 2 mM (0.01% DMSO in a buffer with 50 mM TRIS, pH 8, and 50 mM NaCl).

After an incubation period of 42 h (18 h for the aerobic *S. gordonii*), the growth of microbes was analyzed by visual analysis of the turbidity. MIC represents the lowest concentration without visible turbidity. All experiments were made using independent replicates.

In addition, we monitored the growth of two *P. gingivalis* strains (ATCC 3227 and J374-1) by measuring the OD at 600 nm at different time points (it was not possible to make continuous measurements due to the anaerobic growth conditions of *P. gingivalis*), with the OD_600_ being recorded at 0 h, 16 h, 20 h, 24 h, and 42 h.

## Data availability

The structures presented in this paper have been deposited in the Protein Data Bank (PDB) with the codes 6QQL and 6QRO. All remaining data are contained within the article.

## Conflict of interest

N. T. is employed by PerioTrap Pharmaceuticals GmbH, of which J.-U. R., M. B., S. S., and H.-U. D. are shareholders.

## References

[1] Tonetti M.S., Jepsen S., Jin L., Otomo-Corgel J. (2017). Impact of the global burden of periodontal diseases on health, nutrition and wellbeing of mankind: A call for global action. J. Clin. Periodontol..

[2] Socransky S.S., Haffajee A.D., Cugini M.A., Smith C., Kent R.L. (1998). Microbial complexes in subgingival plaque. J. Clin. Periodontol..

[3] Darveau R.P. (2010). Periodontitis: A polymicrobial disruption of host homeostasis. Nat. Rev. Microbiol..

[4] Kobayashi T., Yoshie H. (2015). Host responses in the link between periodontitis and rheumatoid arthritis. Curr. Oral Health Rep..

[5] Hajishengallis G., Darveau R.P., Curtis M.A. (2012). The keystone-pathogen hypothesis. Nat. Rev. Microbiol..

[6] Hajishengallis G. (2011). Immune evasion strategies of Porphyromonas gingivalis. J. Oral Biosci.

[7] Dominy S.S., Lynch C., Ermini F., Benedyk M., Marczyk A., Konradi A., Nguyen M., Haditsch U., Raha D., Griffin C., Holsinger L.J., Arastu-Kapur S., Kaba S., Lee A., Ryder M.I. (2019). Porphyromonas gingivalis in Alzheimer's disease brains: Evidence for disease causation and treatment with small-molecule inhibitors. Sci. Adv..

[8] Chen Y., Chen X., Yu H., Zhou H., Xu S. (2019). Oral microbiota as promising diagnostic biomarkers for gastrointestinal cancer: A systematic review. OncoTargets Ther..

[9] Laugisch O., Wong A., Sroka A., Kantyka T., Koziel J., Neuhaus K., Sculean A., Venables P.J., Potempa J., Möller B., Eick S. (2016). Citrullination in the periodontium--a possible link between periodontitis and rheumatoid arthritis. Clin. Oral Investig..

[10] Reichert S., Haffner M., Keyßer G., Schäfer C., Stein J.M., Schaller H.G., Wienke A., Strauss H., Heide S., Schulz S. (2013). Detection of oral bacterial DNA in synovial fluid. J. Clin. Periodontol..

[11] Totaro M.C., Cattani P., Ria F., Tolusso B., Gremese E., Fedele A.L., D'Onghia S., Marchetti S., Di Sante G., Canestri S., Ferraccioli G. (2013). Porphyromonas gingivalis and the pathogenesis of rheumatoid arthritis: Analysis of various compartments including the synovial tissue. Arthritis Res. Ther..

[12] Poole S., Singhrao S.K., Kesavalu L., Curtis M.A., Crean S. (2013). Determining the presence of periodontopathic virulence factors in short-term postmortem Alzheimer's disease brain tissue. J. Alzheimers Dis..

[13] Wu Z., Ni J., Liu Y., Teeling J.L., Takayama F., Collcutt A., Ibbett P., Nakanishi H. (2017). Cathepsin B plays a critical role in inducing Alzheimer's disease-like phenotypes following chronic systemic exposure to lipopolysaccharide from Porphyromonas gingivalis in mice. Brain Behav. Immun..

[14] Nie R., Wu Z., Ni J., Zeng F., Yu W., Zhang Y., Kadowaki T., Kashiwazaki H., Teeling J.L., Zhou Y. (2019). Porphyromonas gingivalis infection induces amyloid-β accumulation in monocytes/macrophages. J Alzheimers Dis.

[15] Ide M., Harris M., Stevens A., Sussams R., Hopkins V., Culliford D., Fuller J., Ibbett P., Raybould R., Thomas R., Puenter U., Teeling J., Perry V.H., Holmes C. (2016). Periodontitis and cognitive decline in Alzheimer's disease. PLoS One.

[16] Ishida N., Ishihara Y., Ishida K., Tada H., Funaki-Kato Y., Hagiwara M., Ferdous T., Abdullah M., Mitani A., Michikawa M., Matsushita K. (2017). Periodontitis induced by bacterial infection exacerbates features of Alzheimer’s disease in transgenic mice. NPJ Aging Mech. Dis..

[17] Kamer A.R., Craig R.G., Dasanayake A.P., Brys M., Glodzik-Sobanska L., de Leon M.J. (2008). Inflammation and Alzheimer's disease: Possible role of periodontal diseases. Alzheimers Dement.

[18] Kamer A.R., Dasanayake A.P., Craig R.G., Glodzik-Sobanska L., Bry M., de Leon M.J. (2008). Alzheimer's disease and peripheral infections: The possible contribution from periodontal infections, model and hypothesis. J. Alzheimers Dis..

[19] Smiley C.J., Tracy S.L., Abt E., Michalowicz B.S., John M.T., Gunsolley J., Cobb C.M., Rossmann J., Harrel S.K., Forrest J.L., Hujoel P.P., Noraian K.W., Greenwell H., Frantsve-Hawley J., Estrich C. (2015). Systematic review and meta-analysis on the nonsurgical treatment of chronic periodontitis by means of scaling and root planing with or without adjuncts. J. Am. Dent. Assoc..

[20] Matthews D. (2013). Local antimicrobials in addition to scaling and root planing provide statistically significant but not clinically important benefit. Evid. Based Dent.

[21] Bochtler M., Mizgalska D., Veillard F., Nowak M.L., Houston J., Veith P., Reynolds E.C., Potempa J. (2018). The Bacteroidetes Q-rule: Pyroglutamate in signal peptidase I substrates. Front. Microbiol..

[22] Busby W.H., Quackenbush G.E., Humm J., Youngblood W.W., Kizer J.S. (1987). An enzyme(s) that converts glutaminyl-peptides into pyroglutamyl-peptides. Presence in pituitary, brain, adrenal medulla, and lymphocytes. J. Biol. Chem..

[23] Pohl T., Zimmer M., Mugele K., Spiess J. (1991). Primary structure and functional expression of a glutaminyl cyclase. Proc. Natl. Acad. Sci. U. S. A..

[24] Huang W.L., Wang Y.R., Ko T.P., Chia C.Y., Huang K.F., Wang A.H. (2010). Crystal structure and functional analysis of the glutaminyl cyclase from Xanthomonas campestris. J. Mol. Biol..

[25] Carrillo D.R., Parthier C., Jänckel N., Grandke J., Stelter M., Schilling S., Boehme M., Neumann P., Wolf R., Demuth H.U., Stubbs M.T., Rahfeld J.U. (2010). Kinetic and structural characterization of bacterial glutaminyl cyclases from Zymomonas mobilis and Myxococcus xanthus. Biol. Chem..

[26] Wu V.W., Dana C.M., Iavarone A.T., Clark D.S., Glass N.L. (2017). Identification of glutaminyl cyclase genes involved in pyroglutamate modification of fungal lignocellulolytic enzymes. mBio.

[27] Messer M., Ottesen M. (1964). Isolation and properties of glutamine cyclotransferase of dried papaya latex. Biochim. Biophys. Acta.

[28] Huang K.F., Hsu H.L., Karim S., Wang A.H. (2014). Structural and functional analyses of a glutaminyl cyclase from Ixodes scapularis reveal metal-independent catalysis and inhibitor binding. Acta Crystallogr. D Biol. Crystallogr..

[29] Koch B., Kolenko P., Buchholz M., Carrillo D.R., Parthier C., Wermann M., Rahfeld J.U., Reuter G., Schilling S., Stubbs M.T., Demuth H.U. (2012). Crystal structures of glutaminyl cyclases (QCs) from Drosophila melanogaster reveal active site conservation between insect and mammalian QCs. Biochemistry.

[30] Schilling S., Wasternack C., Demuth H.U. (2008). Glutaminyl cyclases from animals and plants: A case of functionally convergent protein evolution. Biol. Chem..

[31] Wintjens R., Belrhali H., Clantin B., Azarkan M., Bompard C., Baeyens-Volant D., Looze Y., Villeret V. (2006). Crystal structure of papaya glutaminyl cyclase, an archetype for plant and bacterial glutaminyl cyclases. J. Mol. Biol..

[32] Huang K.F., Liu Y.L., Cheng W.J., Ko T.P., Wang A.H. (2005). Crystal structures of human glutaminyl cyclase, an enzyme responsible for protein N-terminal pyroglutamate formation. Proc. Natl. Acad. Sci. U. S. A..

[33] Cynis H., Hoffmann T., Friedrich D., Kehlen A., Gans K., Kleinschmidt M., Rahfeld J.U., Wolf R., Wermann M., Stephan A., Haegele M., Sedlmeier R., Graubner S., Jagla W., Müller A. (2011). The isoenzyme of glutaminyl cyclase is an important regulator of monocyte infiltration under inflammatory conditions. EMBO Mol. Med..

[34] Schilling S., Zeitschel U., Hoffmann T., Heiser U., Francke M., Kehlen A., Holzer M., Hutter-Paier B., Prokesch M., Windisch M., Jagla W., Schlenzig D., Lindner C., Rudolph T., Reuter G. (2008). Glutaminyl cyclase inhibition attenuates pyroglutamate Abeta and Alzheimer's disease-like pathology. Nat. Med..

[35] Nussbaum J.M., Schilling S., Cynis H., Silva A., Swanson E., Wangsanut T., Tayler K., Wiltgen B., Hatami A., Rönicke R., Reymann K., Hutter-Paier B., Alexandru A., Jagla W., Graubner S. (2012). Prion-like behaviour and tau-dependent cytotoxicity of pyroglutamylated amyloid-β. Nature.

[36] Scheltens P., Hallikainen M., Grimmer T., Duning T., Gouw A.A., Teunissen C.E., Wink A.M., Maruff P., Harrison J., van Baal C.M., Bruins S., Lues I., Prins N.D. (2018). Safety, tolerability and efficacy of the glutaminyl cyclase inhibitor PQ912 in Alzheimer's disease: Results of a randomized, double-blind, placebo-controlled phase 2a study. Alzheimers Res. Ther..

[37] Kovacs-Simon A., Titball R.W., Michell S.L. (2011). Lipoproteins of bacterial pathogens. Infect. Immun..

[38] Bender P., Egger A., Westermann M., Taudte N., Sculean A., Potempa J., Möller B., Buchholz M., Eick S. (2019). Expression of human and Porphyromonas gingivalis glutaminyl cyclases in periodontitis and rheumatoid arthritis-A pilot study. Arch. Oral Biol..

[39] Veith P.D., Nor Muhammad N.A., Dashper S.G., Likić V.A., Gorasia D.G., Chen D., Byrne S.J., Catmull D.V., Reynolds E.C. (2013). Protein substrates of a novel secretion system are numerous in the Bacteroidetes phylum and have in common a cleavable C-terminal secretion signal, extensive post-translational modification, and cell-surface attachment. J. Proteome Res..

[40] Gorasia D.G., Veith P.D., Chen D., Seers C.A., Mitchell H.A., Chen Y.Y., Glew M.D., Dashper S.G., Reynolds E.C. (2015). Porphyromonas gingivalis type IX secretion substrates are cleaved and modified by a sortase-like mechanism. PLoS Pathog..

[41] Hutcherson J.A., Gogeneni H., Yoder-Himes D., Hendrickson E.L., Hackett M., Whiteley M., Lamont R.J., Scott D.A. (2016). Comparison of inherently essential genes of Porphyromonas gingivalis identified in two transposon-sequencing libraries. Mol. Oral Microbiol..

[42] Klein B.A., Tenorio E.L., Lazinski D.W., Camilli A., Duncan M.J., Hu L.T. (2012). Identification of essential genes of the periodontal pathogen Porphyromonas gingivalis. BMC Genomics.

[43] Schilling S., Hoffmann T., Wermann M., Heiser U., Wasternack C., Demuth H.U. (2002). Continuous spectrometric assays for glutaminyl cyclase activity. Anal. Biochem..

[44] Schilling S., Manhart S., Hoffmann T., Ludwig H.H., Wasternack C., Demuth H.U. (2003). Substrate specificity of glutaminyl cyclases from plants and animals. Biol. Chem..

[45] Schilling S., Niestroj A.J., Rahfeld J.-U., Hoffmann T., Wermann M., Zunkel K., Wasternack C., Demuth H.U. (2003). Identification of human glutaminyl cyclase as a metalloenzyme. Potent inhibition by imidazole derivatives and heterocyclic chelators. J. Biol. Chem..

[46] Wilks J.C., Slonczewski J.L. (2007). pH of the cytoplasm and periplasm of Escherichia coli: Rapid measurement by green fluorescent protein fluorimetry. J. Bacteriol..

[47] Bickel M., Cimasoni G. (1985). The pH of human crevicular fluid measured by a new microanalytical technique. J. Periodont. Res..

[48] Bickel M., Munoz J.L., Giovannini P. (1985). Acid-base properties of human gingival crevicular fluid. J. Dent. Res..

[49] Baliga S., Muglikar S., Kale R. (2013). Salivary pH: A diagnostic biomarker. J. Indian Soc. Periodontol..

[50] Patel R.M., Varma S., Suragimath G., Zope S. (2016). Estimation and comparison of salivary calcium, phosphorous, alkaline phosphatase and pH levels in periodontal health and disease: A cross-sectional biochemical study. J. Clin. Diagn. Res..

[51] Rajesh K.S., Zareena, Hegde S., Arun Kumar M.S. (2015). Assessment of salivary calcium, phosphate, magnesium, pH, and flow rate in healthy subjects, periodontitis, and dental caries. Contemp. Clin. Dent..

[52] Marsh P.D., McDermid A.S., McKee A.S., Baskerville A. (1994). The effect of growth rate and haemin on the virulence and proteolytic activity of Porphyromonas gingivalis W50. Microbiology (Reading, Engl).

[53] Booth R.E., Lovell S.C., Misquitta S.A., Bateman R.C. (2004). Human glutaminyl cyclase and bacterial zinc aminopeptidase share a common fold and active site. BMC Biol..

[54] Carr P.D., Ollis D.L. (2009). Alpha/beta hydrolase fold: An update. Protein Pept. Lett..

[55] Ruiz-Carrillo D., Koch B., Parthier C., Wermann M., Dambe T., Buchholz M., Ludwig H.H., Heiser U., Rahfeld J.U., Stubbs M.T., Schilling S., Demuth H.U. (2011). Structures of glycosylated mammalian glutaminyl cyclases reveal conformational variability near the active center. Biochemistry.

[56] Holm L., Laakso L.M. (2016). Dali server update. Nucleic Acids Res..

[57] Wilson I. (2011). The Joint Center for Structural Genomics: Exploration of the human gut microbiome. Genome Biol..

[58] Chevrier B., D'Orchymont H., Schalk C., Tarnus C., Moras D. (1996). The structure of the Aeromonas proteolytica aminopeptidase complexed with a hydroxamate inhibitor. Involvement in catalysis of Glu151 and two zinc ions of the co-catalytic unit. Eur. J. Biochem..

[59] Kupski O., Funk L.M., Sautner V., Seifert F., Worbs B., Ramsbeck D., Meyer F., Diederichsen U., Buchholz M., Schilling S., Demuth H.U., Tittmann K. (2020). Hydrazides are potent transition-state analogues for glutaminyl cyclase implicated in the pathogenesis of Alzheimer's disease. Biochemistry.

[60] Benedyk M., Marczyk A., Chruścicka B. (2019). Type IX secretion system is pivotal for expression of gingipain-associated virulence of Porphyromonas gingivalis. Mol. Oral Microbiol..

[61] Ellis T.N., Kuehn M.J. (2010). Virulence and immunomodulatory roles of bacterial outer membrane vesicles. Microbiol. Mol. Biol. Rev..

[62] Schwechheimer C., Kuehn M.J. (2015). Outer-membrane vesicles from Gram-negative bacteria: Biogenesis and functions. Nat. Rev. Microbiol..

[63] Duran-Pinedo A.E., Baker V.D., Frias-Lopez J. (2014). The periodontal pathogen Porphyromonas gingivalis induces expression of transposases and cell death of Streptococcus mitis in a biofilm model. Infect. Immun..

[64] Hayashi J., Hamada N., Kuramitsu H.K. (2002). The autolysin of Porphyromonas gingivalis is involved in outer membrane vesicle release. FEMS Microbiol. Lett..

[65] Bradford M.M. (1976). A rapid and sensitive method for the quantitation of microgram quantities of protein utilizing the principle of protein-dye binding. Anal. Biochem..

[66] Gill S.C., von Hippel P.H. (1989). Calculation of protein extinction coefficients from amino acid sequence data. Anal. Biochem..

[67] Kabsch W. (2010). XDS. Acta Crystallogr. D Biol. Crystallogr..

[68] Winn M.D., Ballard C.C., Cowtan K.D., Dodson E.J., Emsley P., Evans P.R., Keegan R.M., Krissinel E.B., Leslie A.G., McCoy A., McNicholas S.J., Murshudov G.N., Pannu N.S., Potterton E.A., Powell H.R. (2011). Overview of the CCP4 suite and current developments. Acta Crystallogr. D Biol. Crystallogr..

[69] Adams P.D., Afonine P.V., Bunkóczi G., Chen V.B., Davis I.W., Echols N., Headd J.J., Hung L.W., Kapral G.J., Grosse-Kunstleve R.W., McCoy A.J., Moriarty N.W., Oeffner R., Read R.J., Richardson D.C. (2010). PHENIX: A comprehensive Python-based system for macromolecular structure solution. Acta Crystallogr. D Biol. Crystallogr..

[70] Ahrendt K.A., Buckmelter A.J., Grina J., Hansen J.D., Laird E.R., Newhouse B., Ren L., Wenglowsky S.M., Feng B., Malesky K., Mathieu S., Rudolph J., Wen Z., Young W.B., Moreno D.A. (November 11, 2019). Array Biopharma Inc., Genentech Inc., Imidazo[4,5-b]pyridine Derivatives Used as RAF Inhibitors.

