# Supplementary material for: Mammalian-like type II glutaminyl cyclases in Porphyromonas gingivalis and other oral pathogenic bacteria as targets for treatment of periodontitis
Source: J Biol Chem. 2021 Jan 8;296:100263. doi: 10.1016/j.jbc.2021.100263 (PMC7948796; doi:10.1016/j.jbc.2021.100263)
Supplement: Supplementary file 1 — Figures S1 to S7 [file mmc1.pdf]

Supplementary Information for

Mammalian-like type II glutaminyl cyclases in *Porphyromonas gingivalis* and other oral pathogenic bacteria as targets for treatment of periodontitis

Nadine Taudte, Miriam Linnert, Jens-Ulrich Rahfeld, Anke Piechotta, Daniel Ramsbeck, Mirko Buchholz, Petr Kolenko, Christoph Parthier, John A. Houston, Florian Veillard, Sigrun Eick, Jan Potempa, Stephan Schilling, Hans-Ulrich Demuth, Milton T. Stubbs

This PDF file includes:

Supplementary Figure 1: QC activity measurements for bacteroidal QC catalyzed cyclization of the fluorogenic substrate H-Gln-AMC

Supplementary Figure 2: Inhibition of bacteroidal QC activity by metal chelators

Supplementary Figure 3: Inhibition of *Pg*QC activity by 1-benzylimidazole

Supplementary Figure 4: Structure based sequence alignments of bacteroidal QCs, animal QCs and aminopeptidases

Supplementary Figure 5: Structural conservation between bacteroidal QCs and aminopeptidases

Supplementary Figure 6: Overlay of *Bv*QC and *Pg*QC coordinates, demonstrating lack of structure for loops surrounding the active site zinc

Supplementary Figure 7: SDS-PAGE of purified recombinant bacteroidal QCs

Supplementary Movie 1: Structural differences in the *Tf*QC "6-E" loop due to proline *cis-trans* isomerization

Supplementary Movie 2: Sequence conservation in *Pg*QC

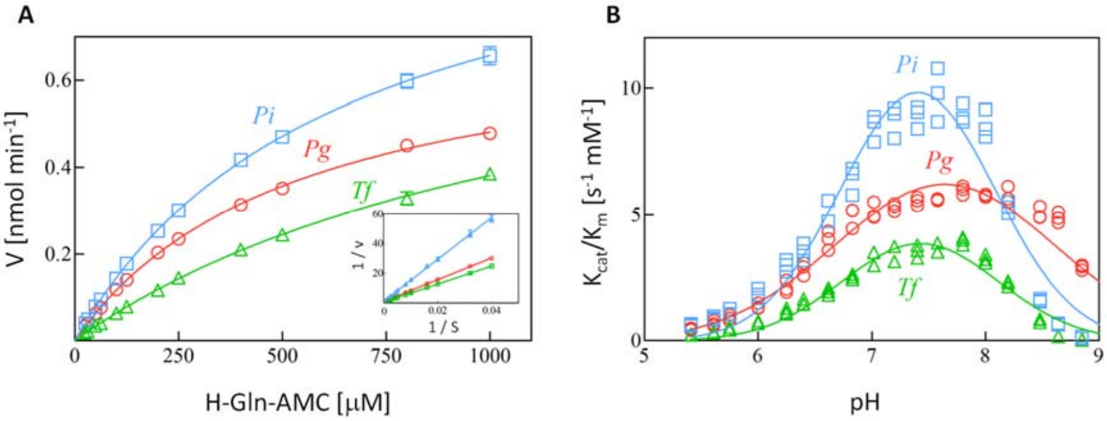

Supplementary Figure 1: QC activity measurements for bacteroidal QC catalyzed cyclization of the fluorogenic substrate H-Gln-AMC. (A) v/S-characteristics and Lineweaver-Burk plots for *Pg*QC (red circles), *Tf*QC (green triangles) and *Pi*QC (blue squares). QC activity measurements were performed in 50 mM Tris-HCl, pH 8.0 and 50 mM NaCl at 30°C. Kinetic data were evaluated using GraFit software (Version 7, Erithacus software Ltd., Horley, UK). **(B)** pH dependence of *Pg*QC, *Tf*QC, and *Pi*QC activities; colors and symbols as in **(A)**. The specificity constants k_{cat}/K_M were determined under first-order rate law conditions with substrate concentrations of 1/10 K_M. Measurements were carried out at 30°C in buffer consisting of 0.1 M Tris-HCl, 0.05 MES, 0.05 mM acetic acid and 0.05 mM NaCl..

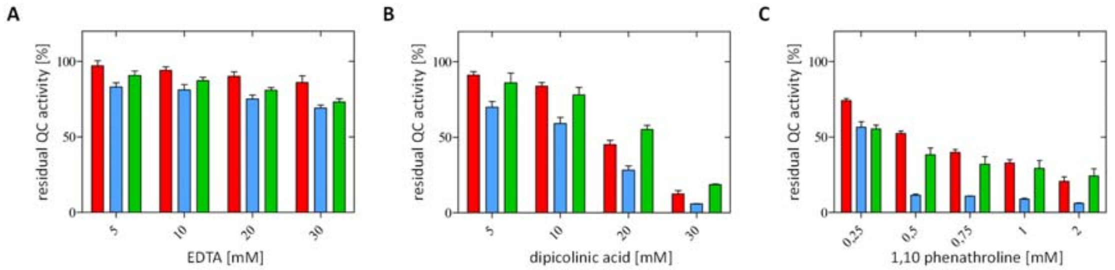

Supplementary Figure 2: Inhibition of bacteroidal QC activity by metal chelators: (A) EDTA, (B) dipicolinic acid and (C) 1,10-phenantroline. 250 µM H-Gln-AMC and increasing chelator concentrations were used to investigate the inhibitory effect on QC activity. Bars indicate residual QC activity following initiation of the reaction by adding 5 nM QC (red: *Pg*QC; green: *Pi*QC; blue: *Tf*QC). All measurements were performed at 30°C as described in the text.

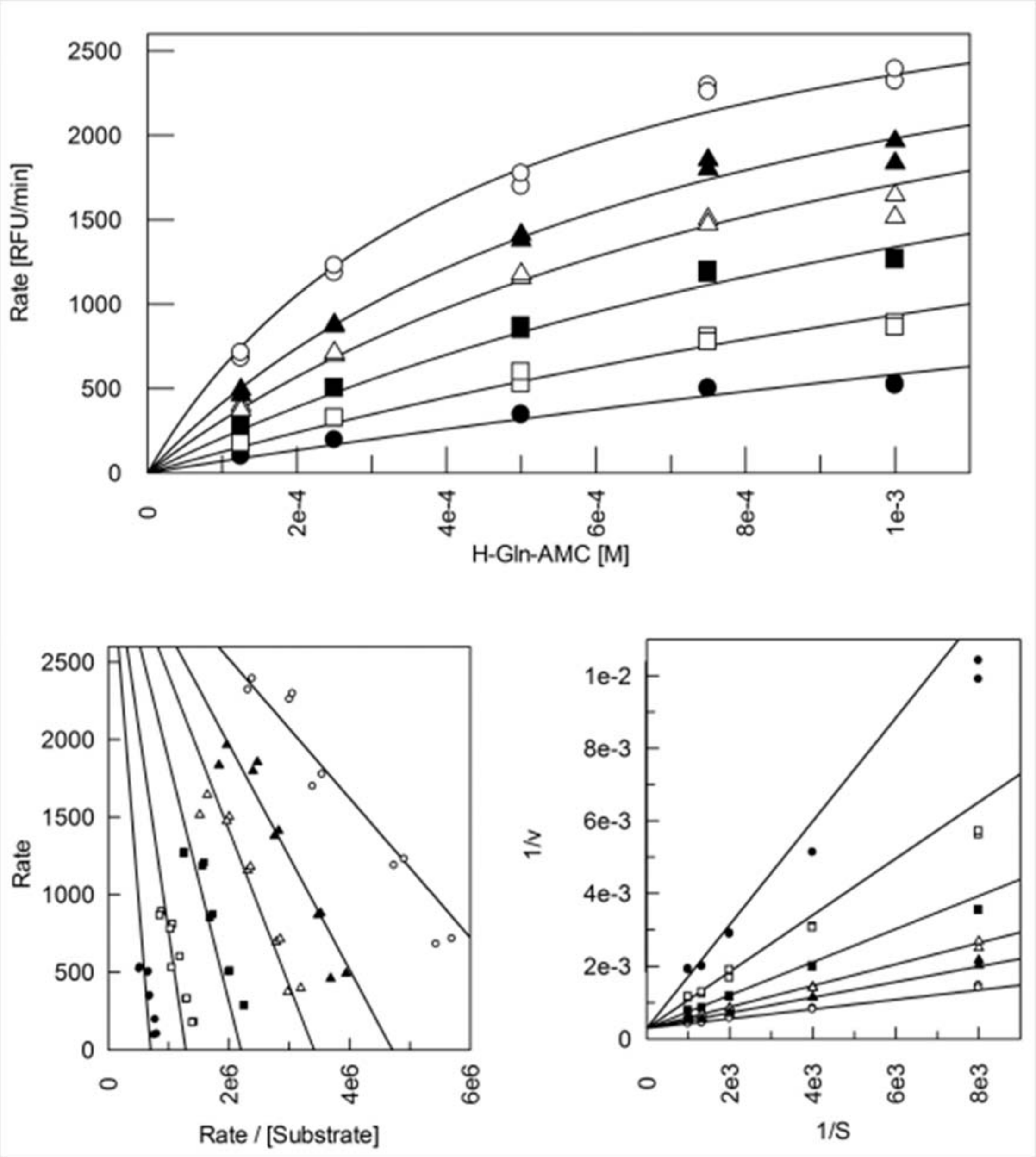

Supplementary Figure 3: Inhibition of *Pg*QC activity by 1-benzylimidazole. v/S characteristics, Lineweaver-Burk and Eadie-Hofstee plots for *Pg*QC catalyzed cyclization of H-Gln-AMC in presence of 1-benzylimidazole. Variation of 1-benzylimidazole concentrations is as follows: (●) 100 µM, (□) 50 µM, (■) 25 µM, (△) 12.5 µM and (▲) 6.25 µM. In each panel, (○) represents reaction without inhibitor. Determinations were carried out in 50 mM Tris-HCl, 50 mM NaCl pH 8.0 and 1% (v/v) DMSO at 30°C.

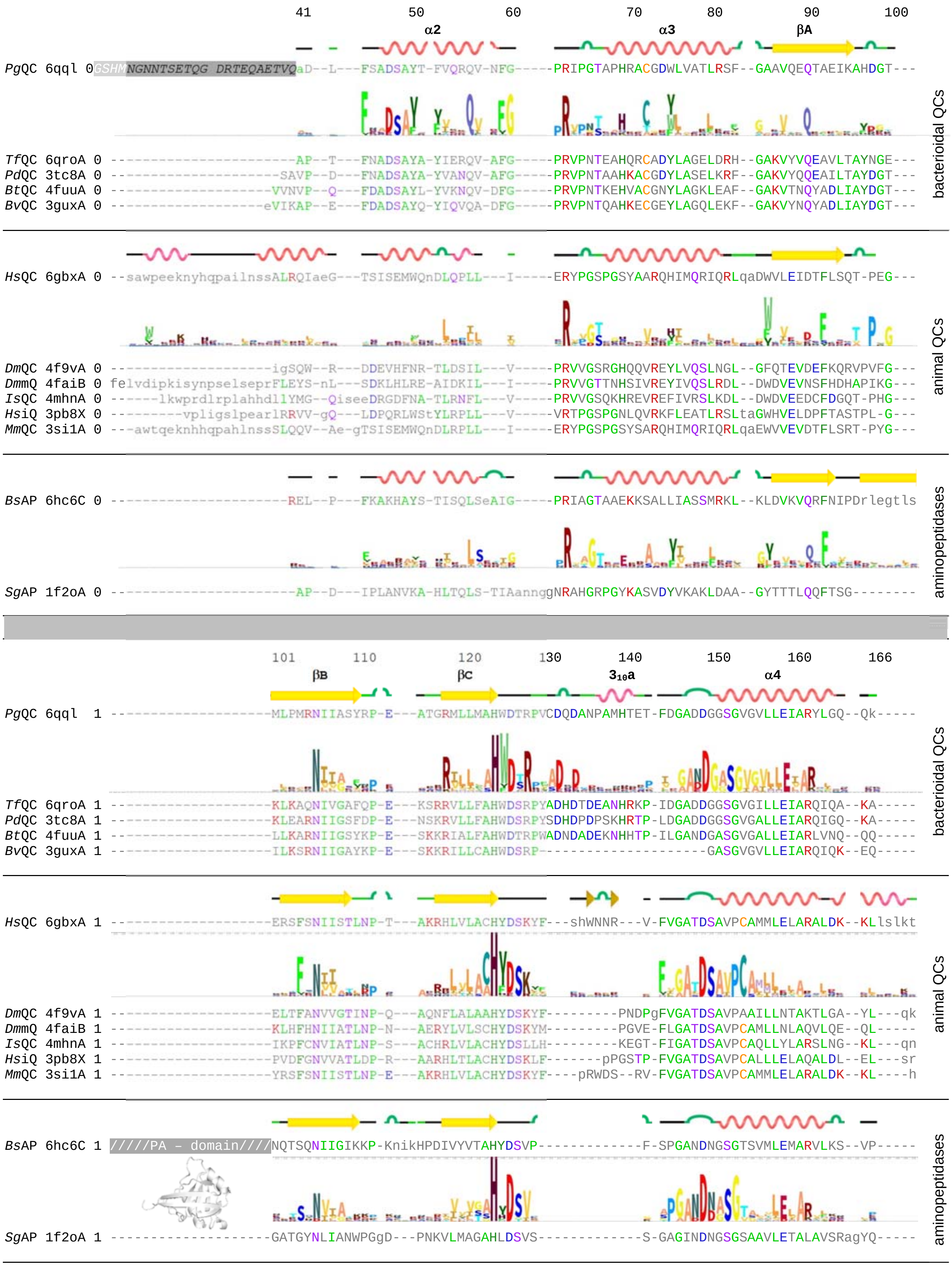

Supplementary Figure 4. Structure based sequence alignments of bacteroidal QCs, animal QCs and aminopeptidases (results from DALI search, residue numbering for *Pg*QC) together with secondary structure assignments and sequence logos (per-residue conservation among related sequences) for *Pg*QC, *Hs*QC and *Bs*AP; the latter exhibits a ca. 120 residue PA-domain insertion. See also **Supplementary Figure 5 and Movie 2**.

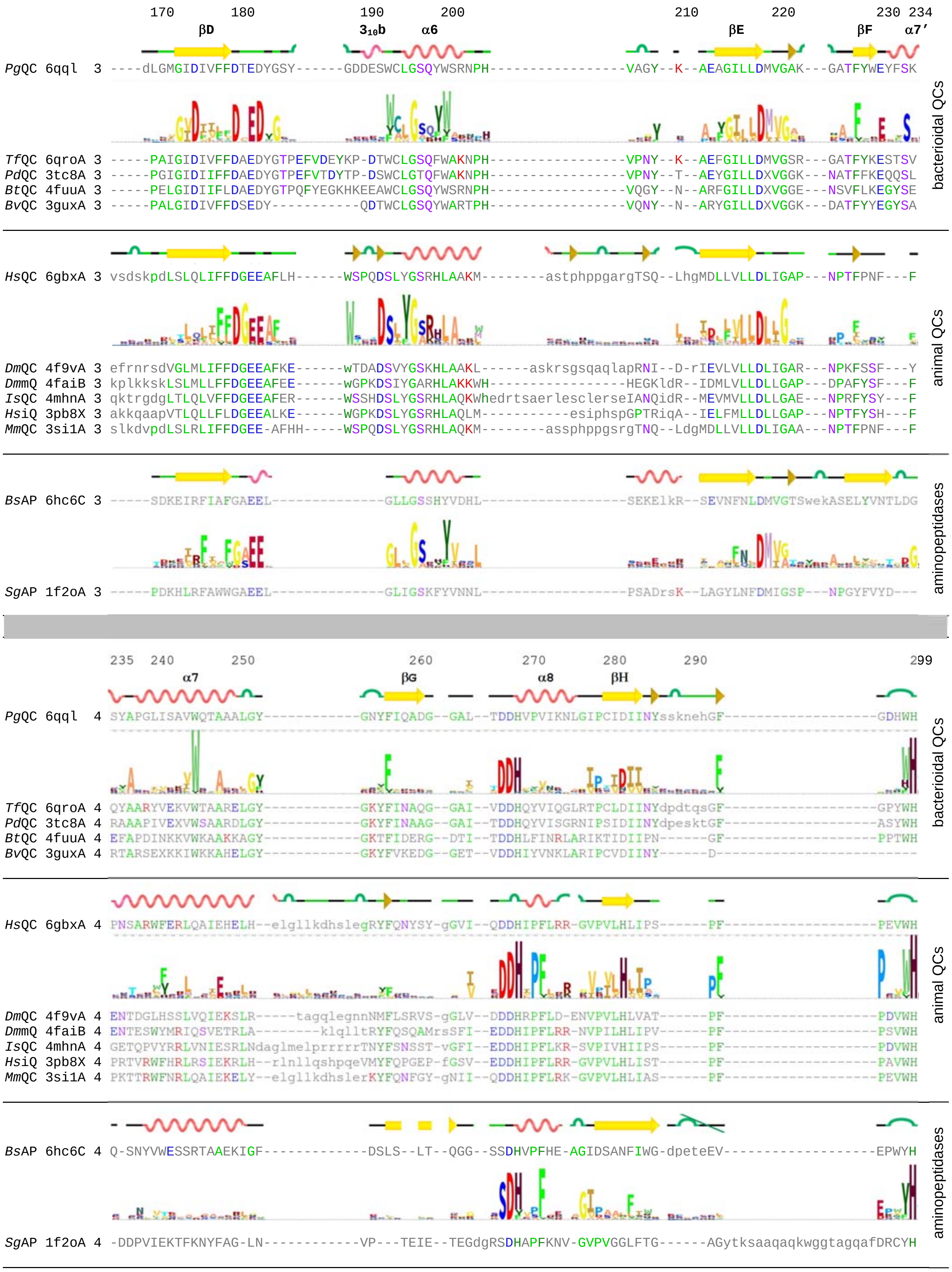

Supplementary Figure 4 (continued). Structure based sequence alignments of bacteroidal QCs, animal QCs and aminopeptidases (results from DALI search, residue numbering for *Pg*QC) together with secondary structure assignments and sequence logos (per-residue conservation among related sequences) for *Pg*QC, *Hs*QC and *Bs*AP; the latter exhibits a ca. 120 residue PA-domain insertion. See also **Supplementary Figure 5 and Movie 2**.

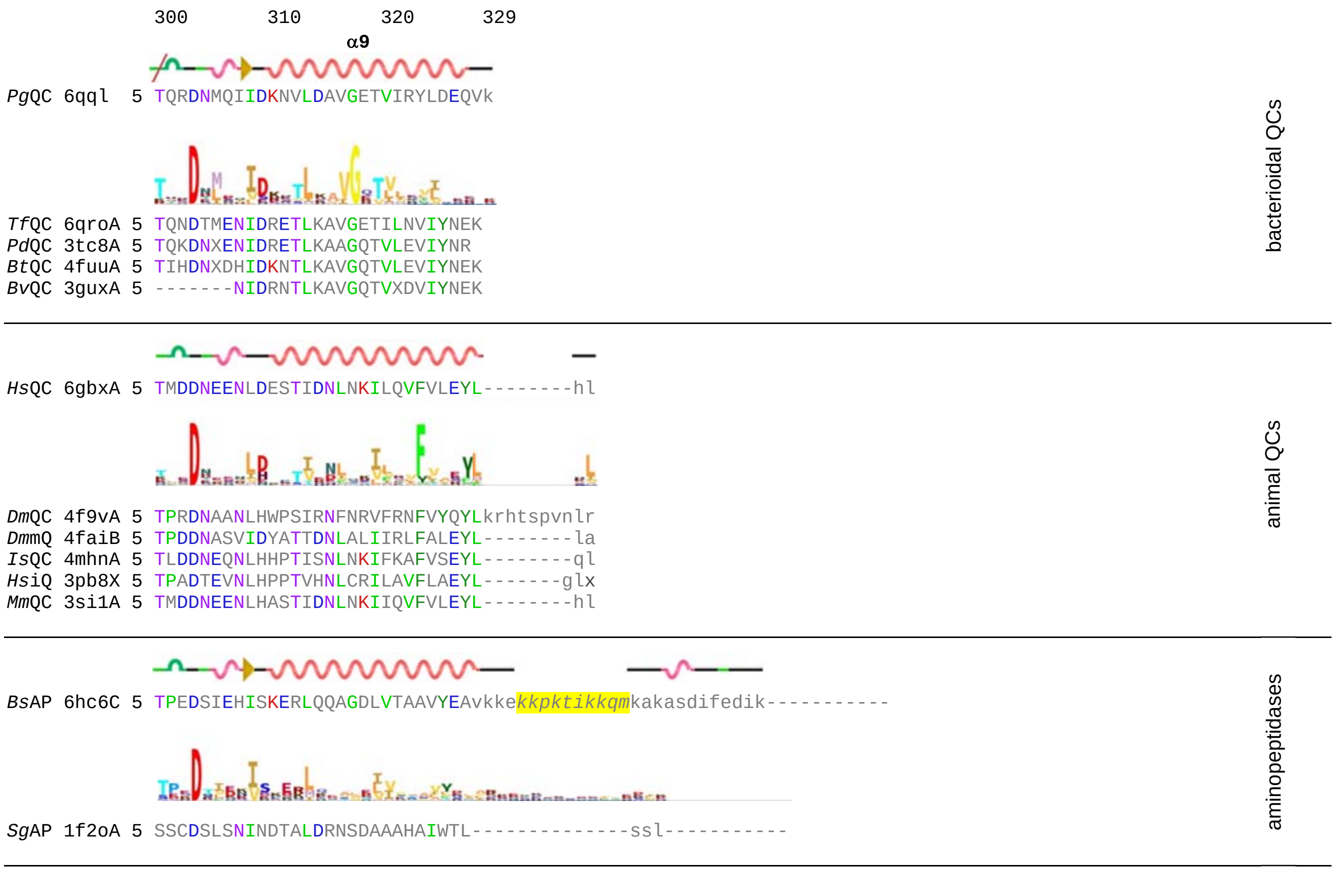

Supplementary Figure 4 (continued). Structure based sequence alignments of bacteroidal QCs, animal QCs and aminopeptidases (results from DALI search, residue numbering for *Pg*QC) together with secondary structure assignments and sequence logos (per-residue conservation among related sequences) for *Pg*QC, *Hs*QC and *Bs*AP; the latter exhibits a ca. 120 residue PA-domain insertion. See also **Supplementary Figure 5 and Movie 2**.

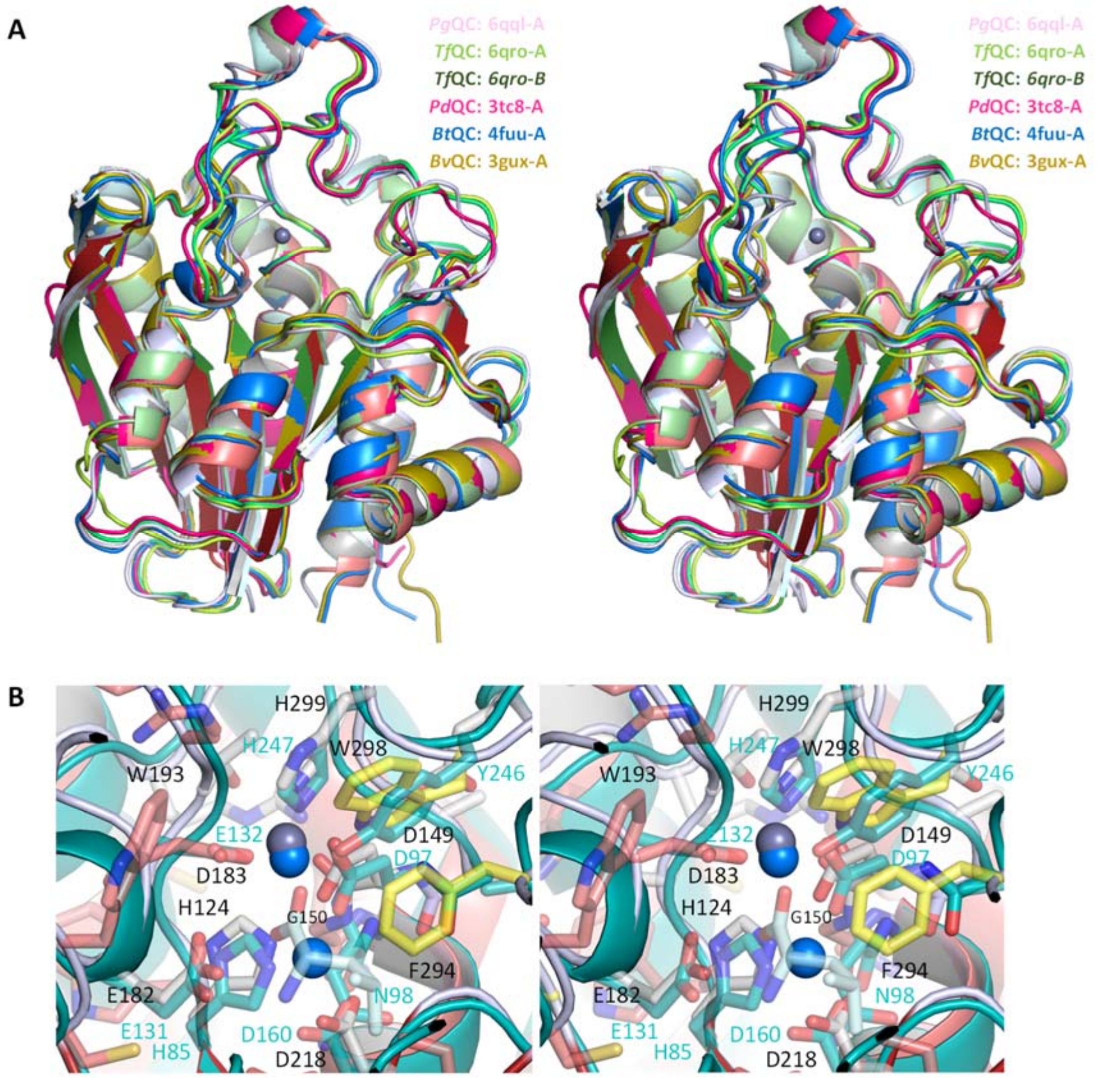

Supplementary Figure 5: Structural conservation between bacteroidal QCs and aminopeptidases. (A) Superposition of bacteroidal QCs in stereo cartoon representation. **(B)** Superposition of *Pg*QC active site (colors as in Supplementary Movie 2) and the *Streptomyces griseus* aminopeptidase (1f2o) with two zinc ions (blue) complexed with L-Leucine product (transparent sticks).

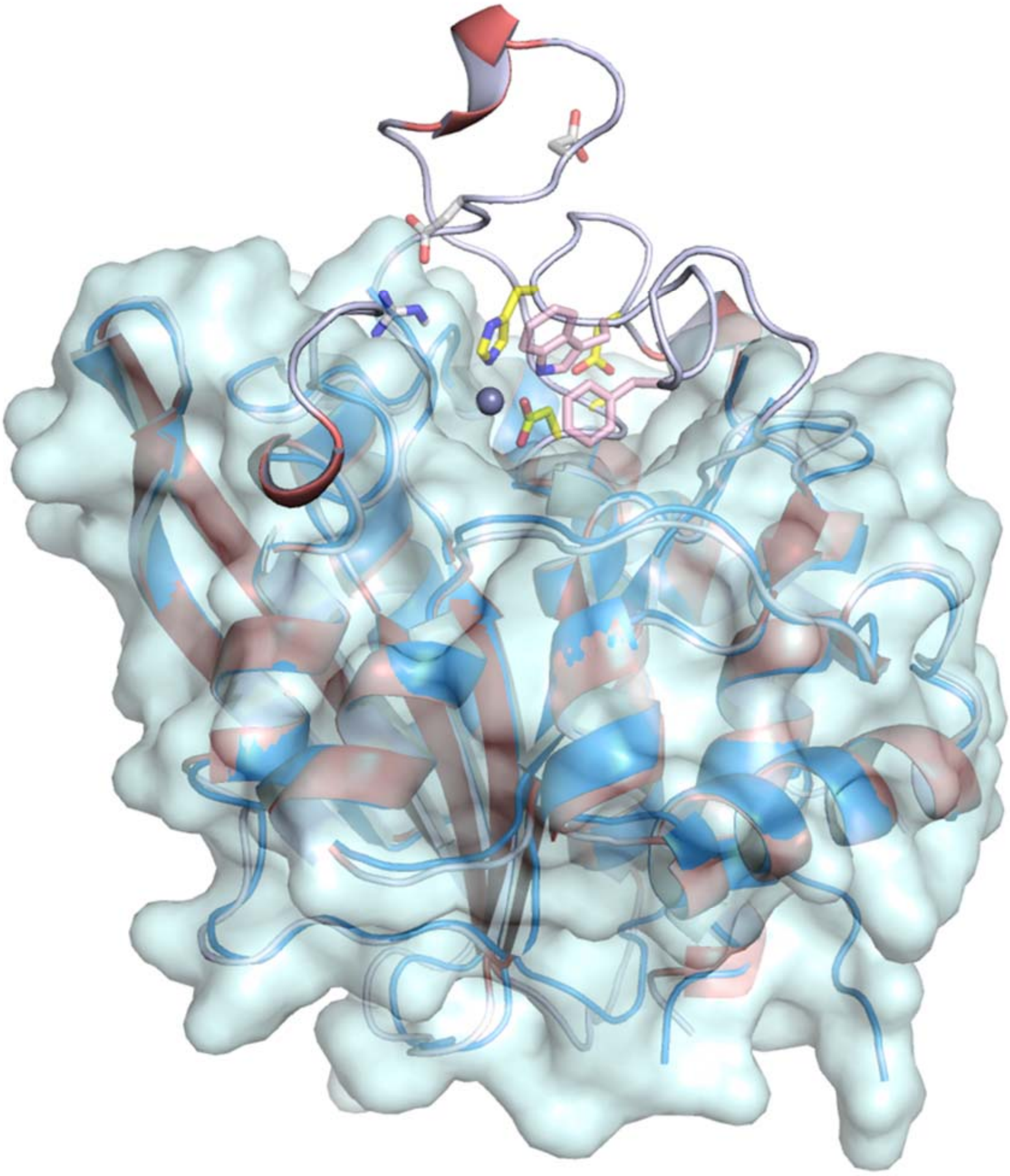

Supplementary Figure 6: Overlay of *Bv*QC (3gux, blue cartoon) and *Pg*QC (6qql, pink cartoon) coordinates, together with blue surface delineating resolved residues in *Bv*QC, demonstrating lack of structure for loops surrounding the active site zinc.

.

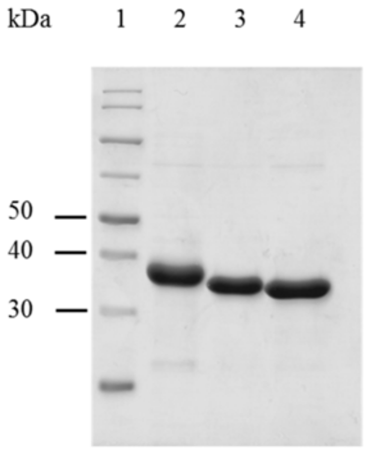

Supplementary Figure 7: SDS-PAGE of purified recombinant bacteroidal QCs expressed in *E. coli* Rosetta(DE3)pLysS. 10 µg purified protein were loaded to a 12% SDS-PAGE and visualized by coomassie staining, lane 1, PageRuler Broad Range unstained (Thermofisher Scientific), lane 2, *Pg*QC, lane 3, *Pi*QC and lane 4, *Tf*QC.

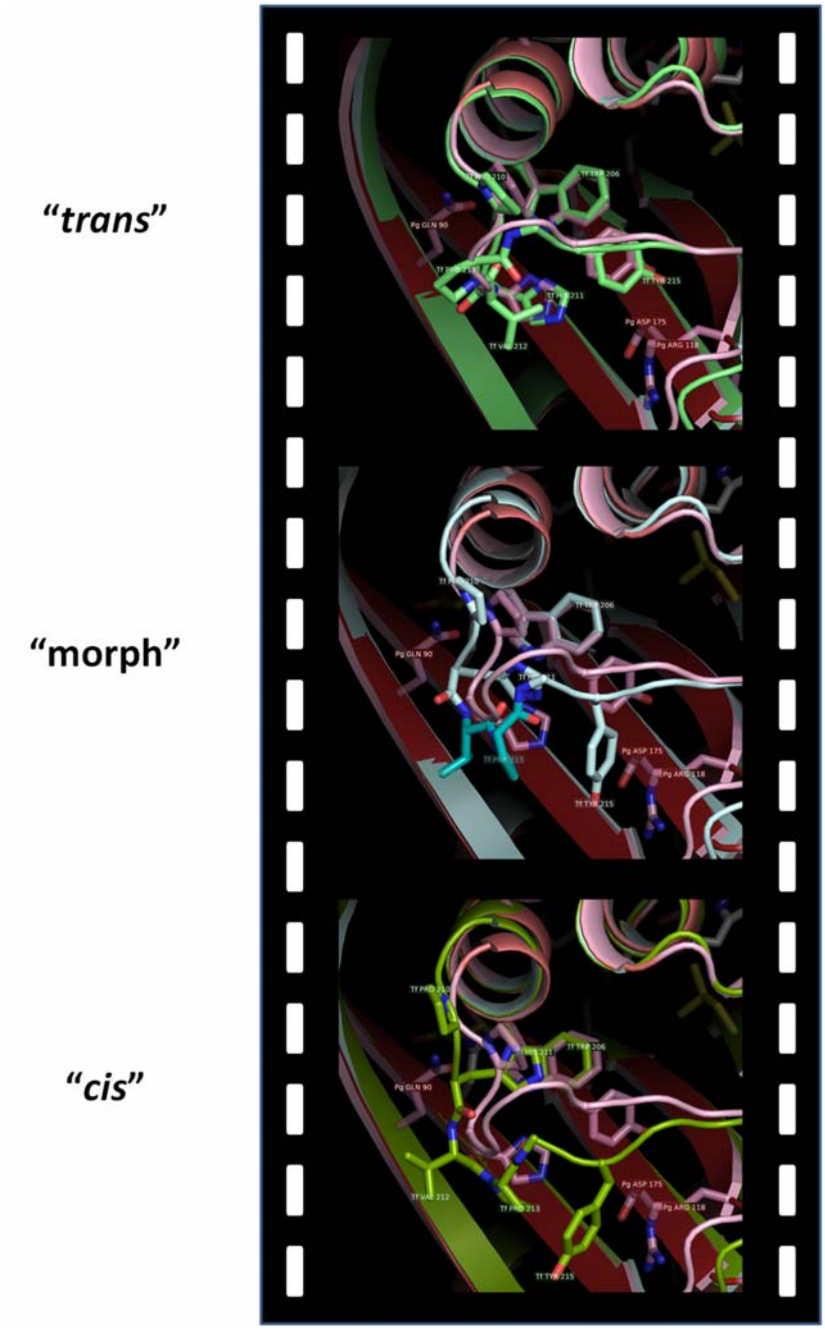

Supplementary Movie 1: Structural differences in the *Tf*QC “6-E” loop due to proline *cis-trans* isomerization. *Tf*QC monomers A (dark green) and B (apple green) superimposed on *Pg*QC (red tones, orientation as in Figure 3C). Due to *cis-*/*trans-* isomerization of the *Tf*Val212-Pro213 peptide bond, two routes are observed for the *Tf*QC “6-E” loop, resulting in a slight reorientation of the α6 helix. Morphing between the two monomer structures was mapped using PyMol. Residues conserved in *Pg*QC-like sequences (Supplementary Figure 4, Movie 2) are shown as pink sticks.

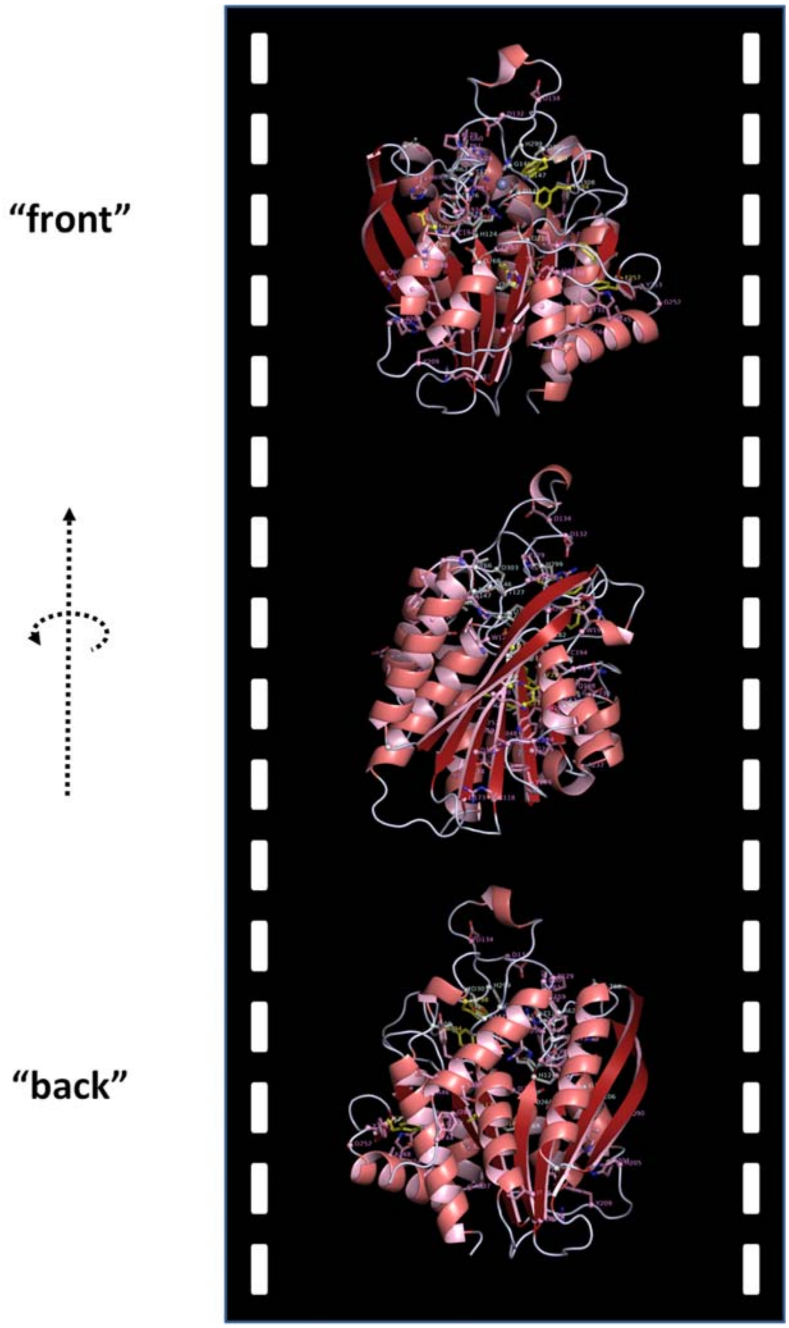

Supplementary Movie 2: Sequence conservation in *Pg*QC. The *Pg*QC cartoon is superimposed with residues conserved among bacteroidal QCs (pink sticks), type II QCs (yellow sticks) and aminopeptidases (white sticks); c.f. Supplementary Figure 4**.**
